# The NarX-NarL two-component system regulates biofilm formation, natural product biosynthesis, and host-associated survival in *Burkholderia pseudomallei*

**DOI:** 10.1038/s41598-021-04053-6

**Published:** 2022-01-07

**Authors:** Mihnea R. Mangalea, Bradley R. Borlee

**Affiliations:** Department of Microbiology, Immunology, and Pathology, Colorado State University, Fort Collins, CO 80523 USA

**Keywords:** Pathogenesis, Molecular biology, Transcriptomics, Microbiology, Bacteria, Biofilms, Microbial genetics, Pathogens

## Abstract

*Burkholderia pseudomallei* is a saprophytic bacterium endemic throughout the tropics causing severe disease in humans and animals. Environmental signals such as the accumulation of inorganic ions mediates the biofilm forming capabilities and survival of *B. pseudomallei*. We have previously shown that *B. pseudomallei* responds to nitrate and nitrite by inhibiting biofilm formation and altering cyclic di-GMP signaling. To better understand the roles of nitrate-sensing in the biofilm inhibitory phenotype of *B. pseudomallei*, we created in-frame deletions of *narX* (Bp1026b_I1014) and *narL* (Bp1026b_I1013), which are adjacent components of a conserved nitrate-sensing two-component system. We observed transcriptional downregulation in key components of the biofilm matrix in response to nitrate and nitrite. Some of the most differentially expressed genes were nonribosomal peptide synthases (NRPS) and/or polyketide synthases (PKS) encoding the proteins for the biosynthesis of bactobolin, malleilactone, and syrbactin, and an uncharacterized cryptic NRPS biosynthetic cluster. RNA expression patterns were reversed in ∆*narX* and ∆*narL* mutants, suggesting that nitrate sensing is an important checkpoint for regulating the diverse metabolic changes occurring in the biofilm inhibitory phenotype. Moreover, in a macrophage model of infection, ∆*narX* and ∆*narL* mutants were attenuated in intracellular replication, suggesting that nitrate sensing contributes to survival in the host.

## Introduction

The ability of many bacteria to substitute nitrate as a terminal electron acceptor in oxygen-limited environments offers various benefits for niche adaptation at comparable free energy changes^1^. Metabolism of N-oxides, inorganic ions comprised of oxygen and nitrogen, derived from exogenous sources in the environment or endogenous metabolic byproducts, preferentially follows the denitrification pathway^2^. Exogenous N-oxides, such as nitrate (NO_3_^−^) and nitrite (NO_2_^−^), can be derived from anthropogenic environmental contamination or host innate immune cells during host–pathogen interactions. *Burkholderia pseudomallei*, an environmental saprophyte^3^ and sapronotic disease agent^4^, is an opportunistic pathogen that transitions between environments where nitrate metabolism can influence bacterial physiology and biofilm dynamics^5^. Biofilms are comprised of extracellular polymeric substances that constitute a protective matrix in which bacteria can reside. The formation and degradation of biofilms is dependent on extracellular cues and intrinsic bacterial signal transduction mechanisms. *B. pseudomallei* 1026b, a clinical isolate^6^, encodes a complete denitrification pathway and responds to exogenous nitrate to some extent by inhibiting biofilm formation and reducing intracellular cyclic di-GMP^5^. The denitrification pathway in *B. pseudomallei* 1026b aligns with the conservation of denitrification genes among the β-proteobacteria, including *B. mallei* and *B. pseudomallei*^7^. Utilizing a transposon library in *B. pseudomallei* 1026b, we have recently characterized a predicted two-component nitrate-sensing system (*narX*-*narL*) that negatively regulates biofilm formation and c-di-GMP production in the presence of nitrate^5^. However, the genetic drivers of biofilm dynamics that link the signal transduction and c-di-GMP turnover to the metabolism of exogenous nitrate in *B. pseudomallei* are poorly understood.

The NarX and NarL proteins comprise a conserved two-component regulatory system whereby NarX responds to nitrate and nitrite ligands to initiate autophosphorylation and activation of the NarL response-regulator receiver domain^8,9^. The *narX-narL* operon (Supplemental Fig. S1A), as initially described in *Escherichia coli*, regulates transcription of gene clusters involved in fermentation and anaerobic respiration^10^ and specifically activates *narGHJI* (membrane-associated nitrate reductase) and *narK* (nitrate/nitrite transporter) promoters to which it is adjacent^9^. The *narX-narL* system has been shown to regulate anaerobic metabolism in *P. aeruginosa*^11^, in which nitrate chemotaxis has recently been demonstrated and hypothesized to play a role in virulence gene regulation^12^. The *narX and narL* genes have recently been implicated in the global regulation of *B. thailandensis* biosynthetic gene clusters via hierarchical control of host survival and anaerobic respiration genes^13^. Saprophytic bacteria, including the *B. pseudomallei* complex, encode biosynthetic NRPS/PKS clusters that are important for survival in the rhizosphere but also may facilitate virulence in eukaryotic hosts^14^. Although we have described nitrate sensing and metabolism in *B. pseudomallei*^5^*,* we know little regarding the genetic regulation of biofilm physiology and the transcriptional regulation associated with nitrate respiration and host survival.

*B. pseudomallei* is a facultative anaerobe that can adapt to oxygen tension in plant-associated rhizospheres^15^ or to an intracellular lifestyle in animal immune cells^16^; environments that are rich in N-oxides and reactive nitrogen intermediates (RNI). Given the broad environmental distribution of *B. pseudomallei*^17^ and its ability to cause severe acute infections in people with no significant medical history^18,19^ or persistent chronic infections in immunocompromised individuals^20,21^, this organism possesses sensing systems to adapt to various niches and extracellular stressors^22^. Analysis of the gene expression profile of *B. pseudomallei* during intracellular infection has revealed a temporal transcriptional adaptation that rapidly modulates bacterial metabolism and physiology inside macrophages^23^. Although stimulated macrophages can inhibit intracellular growth of *B. pseudomallei* through RNI-dependent bactericidal mechanisms, *B. pseudomallei* has the ability to survive and replicate in phagocytes^24^. One approach for identifying resistance mechanisms for RNI is to induce the expression of these mechanisms via sub-lethal levels of the stressor^25^. We used concentrations of nitrate and nitrite that have been previously validated^26,27^, and which we have previously shown to be inhibitory to biofilm formation but not inhibitory to growth^5^, to identify gene loci that are potentially involved in resistance to RNI and likely contribute to intracellular survival of *B. pseudomallei* in host cells.

Here, we describe global transcriptome profiling of gene expression in *B. pseudomallei* under nitrosative stress that is coordinated by the two-component nitrate-sensing system comprised of *narX* and *narL*. RNA-seq analysis of the ∆*narX* and ∆*narL* mutants revealed a global network of genes involved in nitrate/nitrite signal transduction and identified key elements that contribute to biofilm formation, virulence factors, antibiotic resistance markers, as well as the differential regulation of key natural product biosynthetic gene clusters. The nitrosative stress response is alleviated in the absence of either *narX* or *narL*. Additionally, we characterized the intracellular replication of *B. pseudomallei* lacking nitrate-sensing capabilities thereby linking *narX* and *narL* to pathogenicity and survival of this pathogen in the host. These results provide a framework for understanding biofilm inhibition mediated by the nitrosative stress response, linking secondary metabolite biosynthesis and intracellular survival to nitrate metabolism in *B. pseudomallei*.

## Results

### NarX-NarL comprise a nitrate-sensing two-component system in *B. pseudomallei*

Nitrate-sensing two-component systems (TCS) has been described extensively in γ-proteobacteria and in β-proteobacteria, where *Burkholderia* and *Ralstonia* spp. encode the NarX-NarL system, a feature shared with Pseudomonadaceae in the γ-proteobacteria clade but not Neisseriaceae (also β-proteobacteria)^9^. The NarX-NarL TCS is often part of a larger regulon including the dissimilatory nitrate reductase NarGHJI and the transporters/permeases NarK-1 and NarK-2, as is the case in *B. pseudomallei* 1026b (Supplemental Fig. S1A). Analysis of this system via the SMART algorithm (http://smart.embl.de,^28^) revealed a predicted HAMP domain in NarX and a predicted REC domain in NarL (Supplemental Fig. S1B), indicative of two-component signal transduction capabilities^29^. NarX (*E. coli*) is a sensory histidine kinase with a periplasmic sensor domain that is dimeric when bound to its ligand, nitrate (NO_3_^−^)^30^. The NarX (*E. coli*) sensory module (Supplemental Fig. S1C) contains conserved periplasmic domains (P-box and P’-box) that are necessary for ligand binding and response^9^. The HAMP linker-connector-linker domains are common to sensor kinases and are required for signal transduction^29,31^. The *B. pseudomallei* complex (Bpc) which includes *B. mallei* ATCC 23344, an obligate intracellular pathogen, and *B. thailandensis* E264, a non-pathogenic soil saprophyte, share conserved NarX sensory module (Supplemental Fig. S1C) and NarL receiver domain (Supplemental Fig. S1D) sequences with *P. aeruginosa* PAO1 and *E. coli* K12. Additionally, the complete *narX-narL-narGHJI*_*1*_*-narK*_*1*_*-narK*_*2*_ regulon is conserved in its entirety in *B. mallei* ATCC 23344 based on in silico sequence alignment (Supplemental Fig. S2). Collectively, these bioinformatics analyses suggest that the NarX-NarL system functions as an archetypal signal-response regulatory system in *B. pseudomallei* 1026b, and that organisms across the Bpc share this functional genomic element.

### Characterization of *B. pseudomallei* and the NarX-NarL TCS response to nitrate or nitrite in aerobic and anaerobic environments

**Figure 1:**
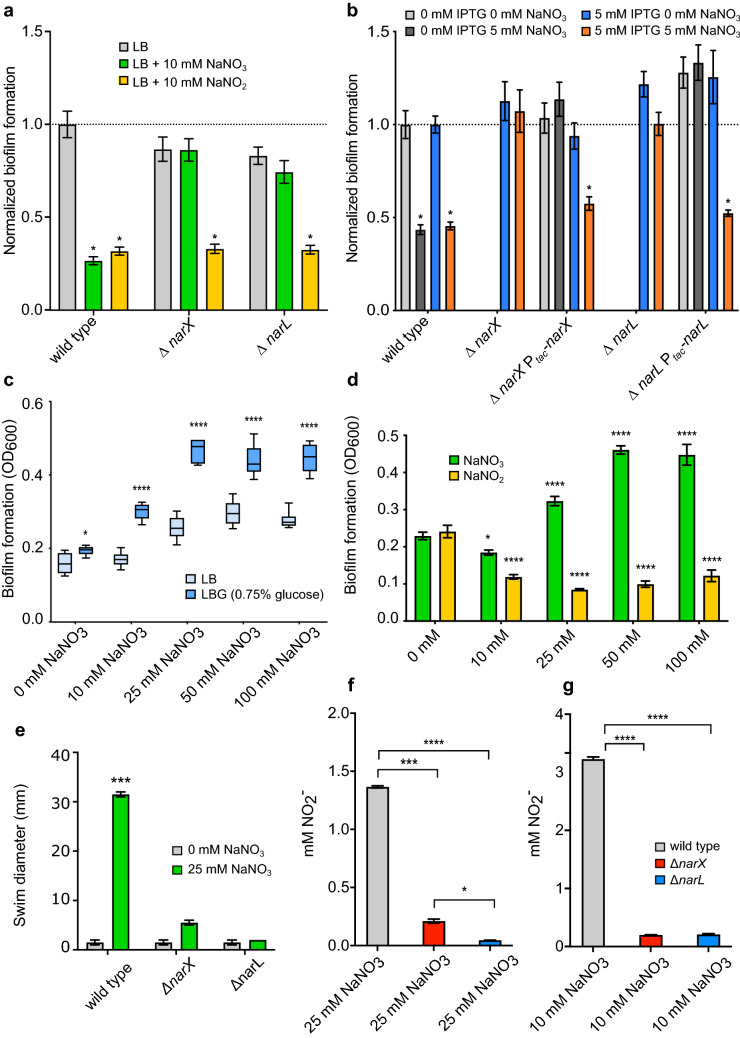
Biofilm formation of *B. pseudomallei* Δ*narX, and* Δ*narL* strains and their complements as compared to the wild type. (**a**) Wild type, Δ*narX*, and Δ*narL* biofilms were grown in LB media (grey) supplemented with either 10 mM NaNO_3_ (green) or 10 mM NaNO_2_ (yellow) at 37 °C for 24 h. (**b**) Complements of Δ*narX* and Δ*narL* mutants were grown in LB media (light grey) supplemented with 5 mM NaNO_3_ (dark grey), 5 mM IPTG (blue), or 5 mM IPTG and 5 mM NaNO_3_ (orange), and compared to wild-type *B. pseudomallei* 1026b containing an empty complementation vector (EV). Asterisks indicate significant differences (*p < 0.0001) calculated with an unpaired Student’s T-test using the Bonferonni method to account for multiple comparisons (n = 12), comparing all groups to the wild type grown in 0 mM IPTG 0 mM NaNO_3_. (**c**) Static growth of wild-type *B. pseudomallei* was measured after 24 h of anaerobic growth at 37 °C with increasing concentrations (0, 10, 25, 50, and 100 mM) of NaNO_3_ in LB media (light blue) or LB media supplemented with 0.75% glucose (dark blue). Asterisks indicate significant differences (*p < 0.05, ****p < 0.0001) calculated with an unpaired Student’s T-test using the Bonferonni method to account for multiple comparisons (n = 12), comparing the LBG group to the LB group across the gradient. (**d**) Static growth of wild-type *B. pseudomallei* was measured after 24 h of anaerobic growth at 37 °C grown in LBG (LB 0.75% glucose) media supplemented with either NaNO_3_ (green) or NaNO_2_ (yellow) at increasing concentrations (0, 10, 25, 50, and 100 mM). Asterisks indicate significant differences (*p < 0.05, ****p < 0.0001) calculated using a Dunnett’s multiple comparison 2-way ANOVA test, comparing both NaNO_3_ and NaNO_2_ groups across the increasing gradient to the 0 mM NaNO_3_ and 0 mM NaNO_2_ dataset. (**e**) Swimming motility of wild type, Δ*narX*, and Δ*narL* strains in 0.3% semi-solid LBG agar (grey) supplemented with 25 mM NaNO_3_ (green) and incubated at 37 °C for 24 h. Asterisks indicate significant differences (***p < 0.001) calculated with an unpaired Student’s T-test using the Bonferonni method to account for multiple comparisons (n = 6), comparing the 25 mM NaNO_3_ condition to the 0 mM NaNO_3_ condition for all strains. Griess reaction results measuring nitrite ion produced by wild type (grey), Δ*narX* (red), and Δ*narL* (blue) strains anaerobically (**f**) after growth in LBG with 25 mM NaNO_3_ or aerobically (**g**) after growth in LB with 10 mM NaNO_3_ at 37 °C for 24 h. Asterisks indicate significant differences (*p < 0.05, ***p < 0.001, ****p < 0.0001) calculated with an unpaired Student’s T-test using the Bonferonni method to account for multiple comparisons (n = 12), where each mutant was compared to the wild type in both conditions.

Previously, we have shown that both sodium nitrate and sodium nitrite inhibit *B. pseudomallei* 1026b biofilm formation in a dose-dependent fashion, implicating five genes (*narX, narL, narH*_*1*_*, narG*_*1*_*,* and *narK*_*2*_*)* in the process^5^. A similar biofilm inhibitory effect via nitrate and nitrite supplementation was observed for the wild type, while both Δ*narX* and Δ*narL* mutants were resistant to nitrate biofilm inhibition but not nitrite (Fig. 1A). Nitrite inhibited biofilm formation in the nitrate sensing-deficient mutants at similar levels to the wild type (Fig. 1A). Nitrate-mediated biofilm inhibition of Δ*narX* and Δ*narL* mutants was restored by complementation of Bp1026b_I1014 (*narX*) and Bp1026b_I1013 (*narL*)) with IPTG-induction and was comparable to the wild type (Fig. 1B). Consistent with our previous transposon insertional mutants^5^, both components of the NarX-NarL system respond to nitrate by biofilm inhibition, however this system is not similarly affected by nitrite at the concentration tested. These results suggest that the NarX-NarL system has specificity for nitrate sensing in the regulation of biofilm dynamics in *B. pseudomallei* 1026b.

To examine the effects of nitrate and nitrite on oxygen-deprived bacterial cells, as commonly found in tissue-associated biofilm^32^ or intracellular infections^33^, we adapted an anaerobic biofilm model for *B. pseudomallei*^34^. Static anaerobic biofilm growth was significantly enhanced with nitrate supplementation with the addition of an added carbon source (0.75% glucose) (Fig. 1C). In contrast to the beneficial effect of nitrate, the addition of sodium nitrite had an inhibitory effect on anaerobic biofilm growth (Fig. 1D). Significant biofilm growth defects were observed starting with the addition of 10 mM NaNO_2_, while subsequent concentration increasingly favored growth in NaNO_3_-supplemented media (Fig. 1D). The observed cessation of growth in the nitrite treatment corresponds to a similar phenotype involving mycobacterial growth repression due to endogenous nitrite accumulation^33^. Interestingly, the addition of exogenous nitrate for anaerobic swim motility assays showed a robust increase in flagellar motility in response to nitrate sensing, considering that absence of both *narX* and *narL* inhibited swimming even with nitrate present (Fig. 1E). These results suggest that *B. pseudomallei* uses nitrate but not nitrite as an alternative terminal electron acceptor during anaerobic biofilm growth, and that nitrite may inhibit anaerobic growth, suggesting hierarchical control during the shift to anaerobiosis.

To analyze relative function of the primary nitrate reductase in *B. pseudomallei* under both aerobic and anaerobic conditions, we next examined the production of nitrite in culture media using the Griess test. Using Griess reagent, which enables colorimetric quantification of nitrite ion in solution, we measured 1.4 mM NO_2_ and 3 mM NO_2_ in anaerobic (Fig. 1F) and aerobic (Fig. 1G) culture conditions, respectively. In both conditions, absence of either *narX* or *narL* significantly inhibited the production of nitrite ion in solution (Fig. 1F,G), indicating that the predicted nitrate-sensing two-component system activates nitrate-nitrite respiration via the primary nitrate reductase in both aerobic and anaerobic conditions. Altogether, these results demonstrate a disparity between exogenous nitrate or nitrite supplementation regarding biofilm growth in *B. pseudomallei*; while both nitrate and nitrite inhibit aerobic biofilm growth, nitrite suppresses anaerobic biofilm growth. Both components of the nitrate-sensing system, *narX* and *narL*, respond to exogenous nitrate in a similar fashion by facilitating nitrate-nitrite respiration, motility, and biofilm inhibition.

### Analyses of global responses to nitrate/nitrite-mediated biofilm inhibition reveals divergent responses

**Figure 2:**
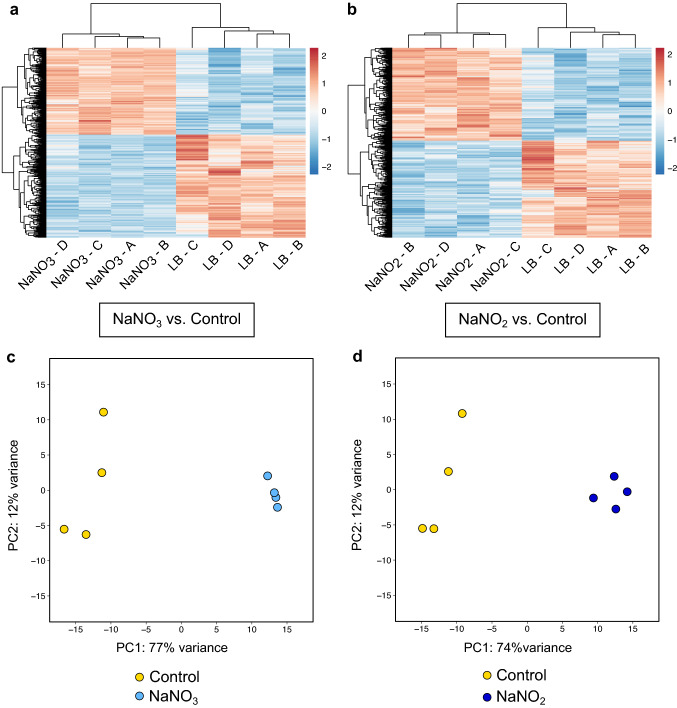
Clustering analyses reveal divergent datasets in the differential expression patterns among wild type samples in both NO_3_^−^ and NO_2_^−^ treatment conditions. Z-score analysis of all differentially regulated transcripts for NaNO_3_ vs. Control (**a**) and for NaNO_2_ vs. Control (**b**). Principal component analysis of entire datasets for NaNO_3_ vs. Control (**c**) and for NaNO_2_ vs. Control (**d**). The control condition is un-supplemented LB media. Hierarchical clustering of all significantly expressed transcripts are presented in panels A and B and principal component analyses represent the complete transcriptomes of all samples as depicted in panels C and D. Principal components were calculated using the regularized log transformation of raw transcript count data in the DESeq2 package. The regularized log-transformed transcript counts from DESeq2 were input into the pheatmap package in R, whereby the data were scaled to Z-scores by rows/genes automatically. Four biological replicates were included for all analyses here.

We next aimed to characterize the global transcriptional response to nitrate/nitrite sensing in *B. pseudomallei* 1026b. Wild type, Δ*narX*, and Δ*narL* strains were grown statically in LB media or supplemented with either 10 mM NaNO_3_ or 10 mM NaNO_2_, revealing divergent transcriptome datasets (Fig. 2). The minimal genetic relatedness between these populations of cells responding to differing environmental conditions is supported by the hierarchical clustering and ordination of rlog-transformed data for each transcript Z-score that is linked by sample and treatment condition (Fig. 2A,B). When comparing wild-type cultures grown in LB supplemented with or without 10 mM NaNO_3_, principal component analysis reveals unrelated populations with 77% of variance explained by one principal component (Fig. 2C). Similar results were observed when comparing wild-type cultures grown in LB supplemented with or without 10 mM NaNO_2_, showing that the first principal component explains 74% of the variance (Fig. 2D). However, when comparing the transcriptome data to account for nitrate and nitrite effects on wild type, most differentially regulated transcripts were affected similarly by nitrate and nitrite (Supplemental Fig. S3). These analyses on the global transcriptome datasets suggest that although substantial differences exist in the response to growth supplemented with either nitrate or nitrite, the majority of the transcripts are similarly upregulated (55%) and downregulated (53%) by both nitrate and nitrite (Supplemental Fig. S3 and Supplementary Table S3). Recently, another group has examined the transcriptional regulation of Δ*narL* in *B. pseudomallei* E8, an environmental isolate, after 30 min of anaerobic growth supplemented with 50 mM NaNO_3_ suggesting that NarL is important for activating electron transport and nitrite reduction to nitric oxide under hypoxic conditions^35^. Here, we present the global expression patterns of *B. pseudomallei* 1026b during aerobic biofilm formation grown with either 10 mM NaNO_3_ or 10 mM NaNO_2_ to gain further insights into the NarX-NarL TCS as biofilms form.

**Figure 3:**
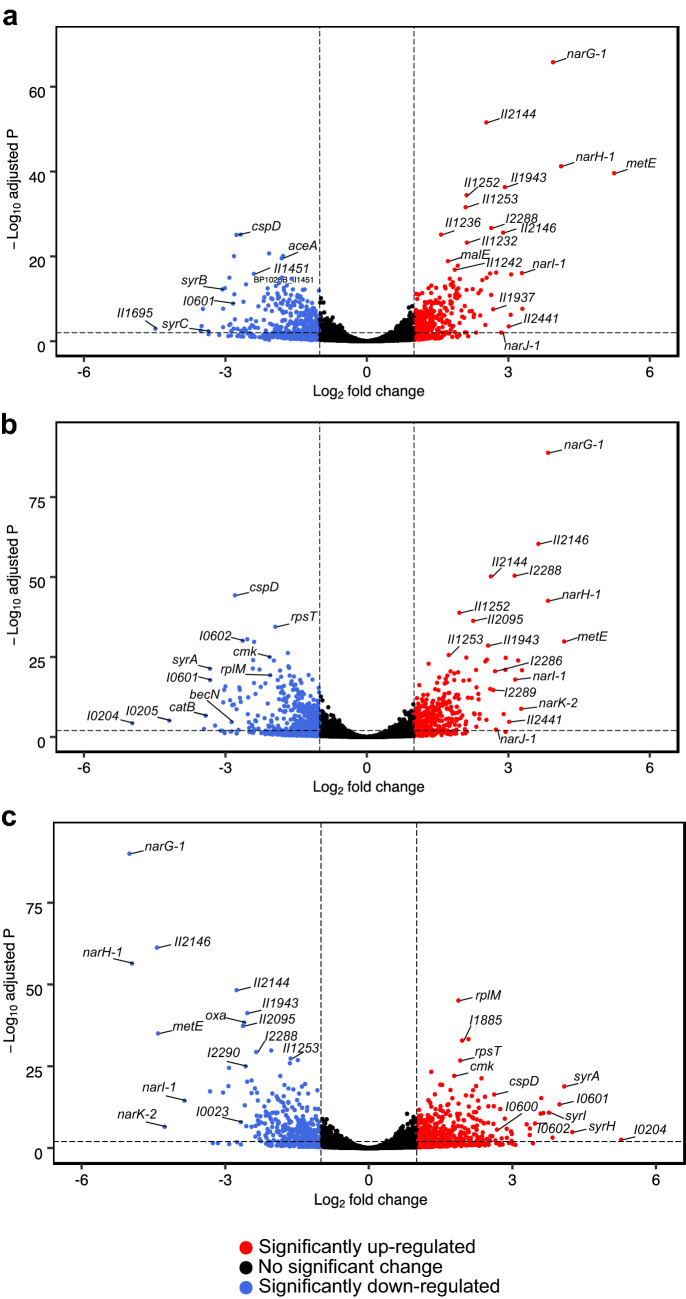
Trends in transcript fold changes in relation to statistical significance for comparably regulated datasets under nitrosative stress are reversed in the Δ*narX* mutant. NO_3_^−^ vs. Control (**a**) and NO_2_^−^ vs. Control (**b**), and Δ*narX* vs. wild type in the NO_3_^−^ condition (**c**). The control condition is un-supplemented LB media. Transcripts are depicted as colored circles; unchanged or middle expression (black), upregulated or high expression (red), downregulated or low expression (blue). Log_2_ fold change values are distributed along the X-axes and Log_10_ adjusted p-values (as determined by the Wald test) are distributed along the Y-axes.

To analyze the global gene expression profiles of wild-type cells grown in the presence of nitrate or nitrite, we used the DESeq2 package for RNA-seq differential expression analysis^36^. At thresholds of log_2_ Fold Change < − 1 or > 1 and adjusted *p*-value < 0.001, 274 genes were up-regulated and 316 genes were down-regulated in the nitrate-supplemented condition versus LB for wild type (Fig. 3A). 237 genes were up-regulated and 271 were down-regulated in the nitrite condition versus LB for the wild type as well (Fig. 3B). However, when comparing the Δ*narX* mutant to the wild type in the nitrate supplemented condition, the trends are reversed (Fig. 3C), indicating that nitrate sensing via NarX is sufficient for a biofilm inhibitory phenotype. The highest and most significantly expressed transcript for both treatment groups as compared to the same transcripts from wild-type *B. pseudomallei* 1026b grown in LB media is Bp1026b_I0767 (*metE*), is a cobalamin-independent methionine synthase that produces methionine from homocysteine^37,38^. *metE* was up-regulated 18.3-fold in the nitrate group and 37.9-fold in the nitrite group (Fig. 3A,B, and Supplementary Table S3). *metE* has been shown to be strongly induced in *R. solanacearum* during plant cell infection^39^ and implicated in homocysteine turnover in *S. typhimurium* during infection where other methionine metabolism enzymes are candidate RNI resistance genes^25,40^.

### Global expression profiles reveal significant differentiation of key functional clusters

**Figure 4:**
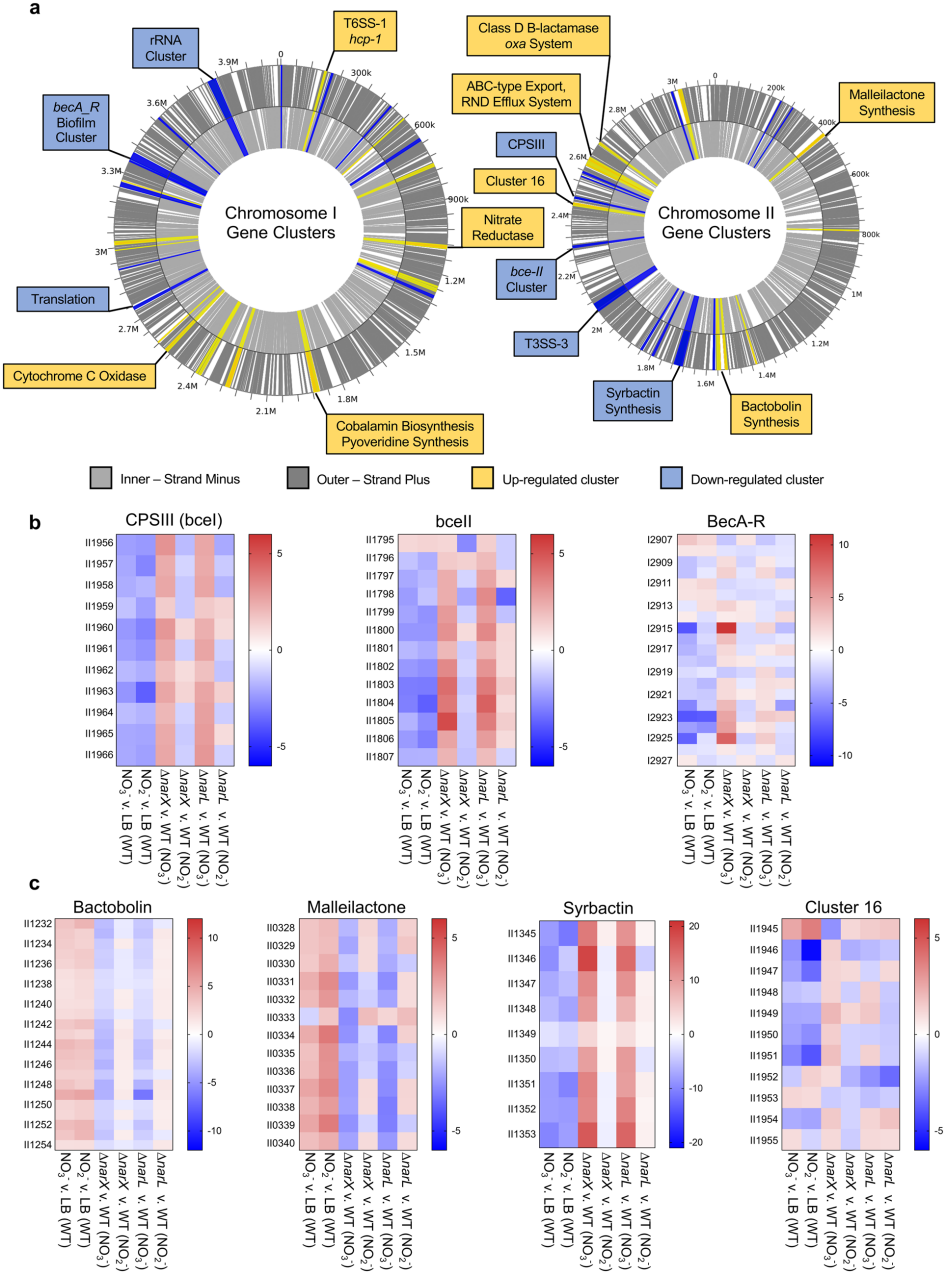
Differentially regulated transcripts comprise clusters of genes distributed across both chromosomes in *B. pseudomallei* 1026b. (**a**) WoPPER analysis reveals biofilm-associated gene clusters, secondary metabolic biosynthetic clusters, general metabolism and respiration, and virulence-associated clusters are differentially regulated in response to nitrate treatment. Up-regulated clusters are highlighted in yellow, and down-regulated clusters in blue. Inner and outer circles represent positive (dark grey) or negative (light grey) strands of DNA. For complete transcriptional analysis of each cluster see Tables S1 and S2. (**b**) Differential expression patterns for key biofilm-associated gene clusters, CPSIII/*bce-I*, *bce-II*, *becA-R*. (**c**) Differential regulation of secondary metabolite synthesis clusters, bactobolin, malleilactone, syrbactin, and the cryptic cluster 16. Data was extracted from DESeq2 analysis of significantly regulated gene loci and pooled as mean fold change values, which are visualized here. Uniform regulation is evident among the treatment conditions, with nitrate and nitrite conditions similarly regulating clusters opposed by Δ*narX* and Δ*narL* mutant trends in the nitrate treatment condition. Color density gradients represent up-regulation (red), down-regulation (blue), and no differential regulation (white).

Using the Webserver for Position Related data analysis of gene Expression in Prokaryotes (WoPPER)^41^, we identified key disparities regarding the effects of nitrate and nitrite on the differential regulation of metabolic, respiratory, biofilm, virulence, and biosynthetic gene clusters in *B. pseudomallei* 1026b (Fig. 4). By identifying and mapping physically contiguous clusters of genes along both chromosomes that respond similarly to nitrate, we provide a visual validation of the differential expression statistical output provided by DESeq2. Several transcripts from these clusters were chosen for additional validation of expression via quantitative real-time PCR: I1018 (*narG-1*), II1965 (CPSIII), I1913 (*relA*), II1245 (bactobolin), II1353 (syrbactin), I0189 (*hcp-1*) (Supplemental Fig. S4). This graphical representation of the gene expression data set highlights important biofilm-associated clusters, antibiotic resistance-associated clusters, and interestingly, the localization of secondary metabolite biosynthetic gene clusters on chromosome II (Fig. 4A). In total, WoPPER analysis identified 27 clusters on chromosome I (Supplementary Table S1) and 21 clusters on chromosome II (Supplementary Table S2) as differentially regulated in response to exogenous nitrate.

Not surprisingly, among the most highly expressed transcripts in both nitrate and nitrite treatment groups compared to LB were genes in the *narXL-narGHJI-narK*_*2*_*-narK*_*1*_ regulon. Bp1026b_I1015 (*narI-1*), I1016 (*narJ-1*), I1017 (*narH-1*), I1018 (*narG-1*), I1019 (*narK-2*), and I1020 (*narK-1*) were collectively up-regulated at mean fold change of 9.7 in the nitrate treatment group, and at mean fold change of 10.5 in the nitrite group. Among these loci, the α- and β-subunits of the dissimilatory nitrate reductase *narG-1*, and *narH-1*, respectively, were the most highly and significantly expressed in the nitrate and nitrite groups (Supplementary Table S3). Relative abundance of the *narG-1* transcript expressed in both nitrate and nitrite-supplemented growth conditions measured by quantitative real-time PCR supported the observed transcript expression levels in the RNA-seq dataset (Supplemental Fig. S4).

Our analysis also revealed significant downregulation of several gene clusters associated with polysaccharide biosynthesis. The matrix of extracellular polymeric substances (EPS) in *Burkholderia* spp*.* encompasses numerous polysaccharides, adhesins, lipids, and extracellular DNA that contribute to aggregation and biofilm formation^42^. This analysis revealed significant downregulation of capsular polysaccharides CPS III/*bce-I* (Bp1026b_II1956 – Bp1026b_II1966, *bce-II* (Bp1026b_II1795 – Bp1026b_II1807)^42^, and *becA-R* (Bp1026b_I2907 – Bp1026b_I2927)^43^ biofilm-associated biosynthesis gene cluster, among the differentially expressed datasets from both the nitrate and nitrite conditions (Fig. 4B). Altogether, these results demonstrate the transcript level reduction of key EPS matrix components, notably exopolysaccharide clusters, which correlate with the biofilm inhibition response to both nitrate and nitrite supplementation in *B. pseudomallei*. A representative transcript of CPS III, II1965, was significantly reduced in expression in both nitrate and nitrite-supplemented growth conditions when measured by quantitative real-time PCR (Supplemental Fig. S4). The reduction of expression in these biofilm-associated clusters is also dependent on the intact NarX-NarL system, as all the above trends are reversed in pairwise comparisons of Δ*narX* (Fig. 3C), and to Δ*narL* (Supplementary Table S3) to the wild type under nitrate treatment.

In addition to polysaccharide biosynthetic clusters, *Burkholderia* spp. encode several gene clusters that produce antimicrobial natural products^44^. *B. pseudomallei* 1026b encodes a combination of 15 nonribosomal peptide synthetase (NRPS) and polyketide synthase (PKS) biosynthetic gene clusters residing on both chromosomes^45^. Our analysis identified four clusters on chromosome II in which all loci were similarly differentially regulated in both the nitrate and nitrite stress comparisons (Fig. 4B). Bactobolin is an antibiotic encoded by a 120-Kb DNA element in *B. pseudomallei* and *B. thailandensis* that is responsive to AHL mediated quorum sensing^46^. The bactobolin biosynthetic cluster, encoded by loci Bp1026b_II1232 – Bp1026b_II1251 (*btaA–btaU*)^47^, was markedly upregulated at a mean fold change of 3.1 in the nitrate treatment group and 3.0 in the nitrite treatment group. In both comparisons, all gene loci in the bactobolin cluster were among the most significantly expressed with consistently low false discovery rates.

Another biosynthetic gene cluster that was identified as significantly upregulated in both nitrate and nitrite stress conditions encodes for malleilactone, a cytotoxic siderophore^48^. The malleilactone biosynthetic cluster contains 13 ORFs (Bp1026b_II0330–Bp1026b_II0341), including two large polyketide synthase gene loci (*malA* and *malF*) and a LuxR-type transcription factor (*malR*), indicating that its expression is mediated by an AHL quorum-sensing system^48^. When comparing wild type in the nitrate-supplemented media versus the control media, 7/13 of the malleilactone genes were significantly upregulated by a mean fold change ratio of 2.4, and in the nitrite condition versus the control media, 11/13 loci are significantly expressed at a fold change ratio of 3.1. Interestingly, malleilactone is among the few PKS/NRPS clusters that is conserved among the Bpc, including *B. mallei* and *B. thailandensis*, the latter of which has been shown to be important for virulence^45,48^. Thus, in apparent coordination with bactobolin antibiotic expression, *B. pseudomallei* similarly regulates the transcription of the malleilactone siderophore biosynthetic genes as a response to the biofilm-inhibitory doses of nitrate and nitrite (Fig. 4B).

In stark contrast to bactobolin and malleilactone upregulation under these conditions, a cluster of genes from Bp1026b_II1345–Bp1026b_II1353 (*syrA–syrI*) encoding syrbactin^45^, was significantly downregulated in both the nitrate and nitrite treatment groups. Syrbactin is a proteasome inhibitor produced by a hybrid PKS/NRPS cluster comprised of nine genes. All nine genes in the syrbactin cluster were downregulated at a mean fold change of -7.1 in the nitrate treatment group and -7.5 in the nitrite treatment group. For both treatment groups, syrbactin biosynthetic cluster genes were among the most downregulated genes in the differential expression analysis. However, analysis of Δ*narX* and Δ*narL* under nitrate stress compared to the wildtype reversed this trend, implicating the NarX-NarL system in regulation of secondary metabolism in *B. pseudomallei* 1026b (Fig. 4B). Representative transcripts from bactobolin (II1245) and syrbactin (II1353) were also inversely regulated in both nitrate and nitrite-supplemented growth conditions as measured by quantitative real-time PCR (Supplemental Fig. S4). Further supporting this hypothesis, a homolog of the recently described global regulator of secondary metabolism^49^*,* Bp1026b_I0582 (*scmR*), is significantly downregulated -2.0-fold in the nitrate treatment group and -1.7-fold in the nitrite group. ScmR is a LysR-type transcriptional regulator that represses many biosynthetic gene clusters in the Bpc^49^. In both analyses of Δ*narX* and Δ*narL* under nitrate stress, *scmR* is notably upregulated, 2.6- and 2.9-fold, respectively (Supplementary Table S3). Collectively, these results suggest that nitrosative stress induces transcription of bactobolin antibiotic and malleilactone siderophore biosynthesis and represses transcription of the syrbactin proteasome inhibitor via the global regulator *scmR,* which is dependent on a functioning NarX-NarL two-component system.

### *B. pseudomallei* nitrate sensing mutants are deficient in intracellular survival in mouse macrophages

**Figure 5:**
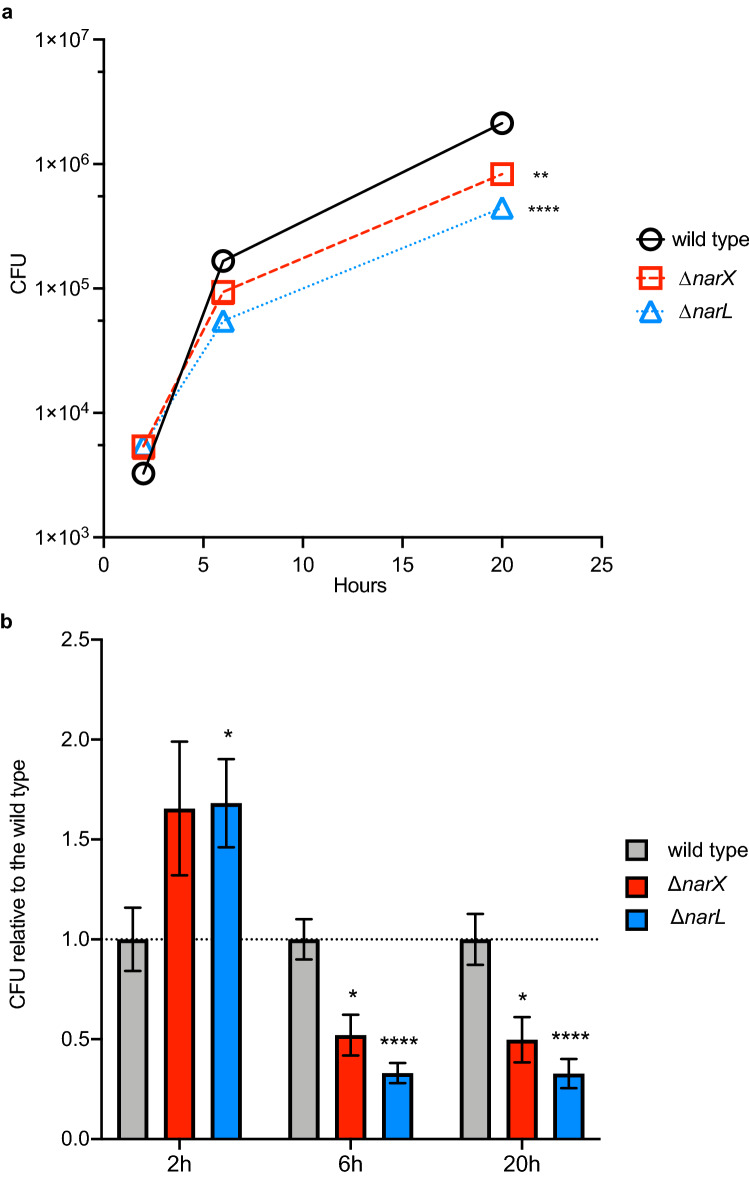
*B. pseudomallei* mutants lacking NarX and NarL are deficient in intracellular replication despite being internalized at a higher rate than the wild type. RAW264.7 cell monolayers were infected at an MOI of ~ 2 with three strains (wild type, ∆*narX*, and ∆*narL*) and intracellular survival was measured at 2 h, 6 h, and 20 h post-infection. At 1 h post-infection, cells were treated with 750 μg/mL kanamycin to kill extracellular bacteria. Total CFU levels were calculated (**a**) as well as CFU relative to the wild type (**b**) at all time-points. The internalization efficiency of the mutant is evident at 2 h post-infection, yet the intracellular replication efficiency is hindered at 6 h and infection is attenuated at 20 h (**b**). Wild type is depicted in black (**a**) or grey (**b**), with ∆*narX* in red and ∆*narL* in blue throughout. Statistical significance was determined using the Holm-Sidak method across multiple Student’s T-tests (*p < 0.05, **p < 0.01, ****p < 0.001), whereby each mutant is compared to the wild type at all time points tested.

Successful intracellular pathogens require the ability to survive within phagocytic cells such as macrophage that bombard intruders with reactive oxygen and nitrogen intermediates^16,24^. To determine the ability of the nitrate sensing-deficient mutants to infect and survive in a hostile macrophage environment amid nitrosative stress, a murine macrophage cell line (RAW 246.7) was co-incubated with wild-type, Δ*narX,* and Δ*narL* strains at a MOI of ~ 2. The number of intracellular bacteria was determined after 2 h, 6 h, and 20 h post-exposure when eukaryotic cells were lysed and plated to count bacterial colony forming units (CFU). At 2 h, there was a notable increase in bacterial internalization into the murine macrophage cells; however, at 6 h and 20 h Δ*narX* and Δ*narL* mutants were significantly impaired at intracellular survival (Fig. 5A). Two hours after infection, the wild type was recovered at lower titers than either of the two mutant strains (Fig. 5B). Δ*narX* and Δ*narL* were internalized at 166% and 168% compared to the wild type, respectively. At 6 h post-infection, Δ*narX* colony forming units were recovered at 56%, and Δ*narL* at 33% compared to the wild type, indicating an early-onset defect in intracellular replication. After 20 h of infection, the observed Δ*narX* and Δ*narL* defects were maintained and CFU were recovered at 51% and 33% compared to wild type, respectively (Fig. 5A). When comparing Δ*narX* and Δ*narL* mutants to wild type infection dynamics, CFU values were statistically significant at all three time-points of infection, however Δ*narL* was more drastically attenuated in this model. Thus, these results indicate that although attachment and internalization of the mutant strains tested are elevated at 2 h post-infection, there are significant defects in *B. pseudomallei* lacking the NarX sensor kinase and the NarL DNA-binding regulator during intracellular replication up to 20 h post-infection.

## Discussion

**Figure 6:**
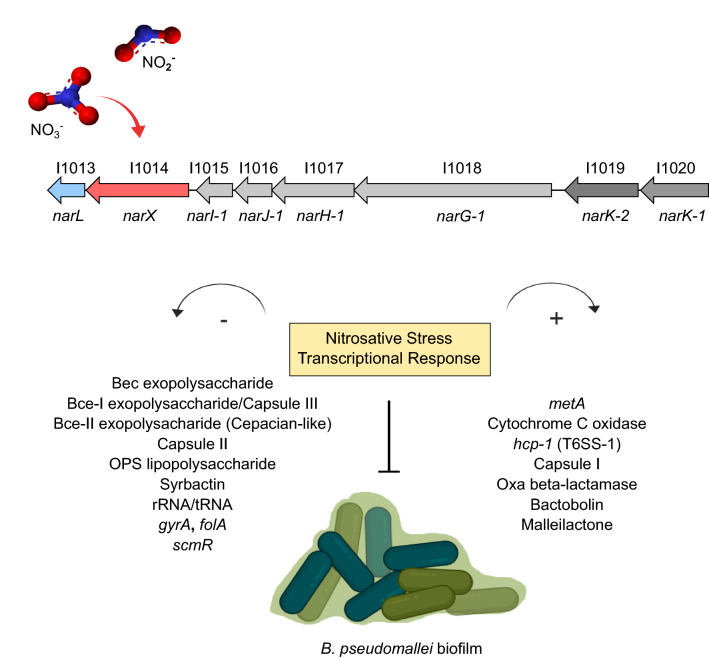
Model of nitrate-dependent biofilm inhibition and transcriptional response in *B. pseudomallei* 1026b. Nitrate, and to a lesser extent nitrite, activate the *narXL* sensing mechanism in *B. pseudomallei* which leads to metabolism of N-oxides, activation of a nitrosative stress response and indirect inhibition biofilm formation. Several biofilm-associated gene clusters such as capsules, exopolysaccharides, and lipopolysaccharides are down-regulated along with several housekeeping genes that are necessary for growth and division. Conversely, secondary metabolic biosynthesis clusters are up-regulated in conjunction with pathogenicity-associated genes, an alternative respiration mechanism, as well as the methionine metabolism gene *metE*. The biofilm model figure was created with BioRender.com.

Understanding the biofilm dynamics of *B. pseudomallei* in the context of environmental sensing of nitrate and nitrite has broad implications for transmission epidemiology as well as the pathogenicity and clinical mitigation of this facultative intracellular organism. The metabolic versatility and adaptability of *B. pseudomallei* underscores its success as a ubiquitous tropical saprophyte^17^ as well as a human pathogen capable of infecting all tissue types tested^50^. *B. pseudomallei* is recognized as a potential threat to people who come in contact with contaminated soil from domestic gardens in endemic regions^51,52^, and may be linked to anthropogenic sources of nitrate and urea in these gardens^53,54^. The present study characterizes the nitrosative stress response in *B. pseudomallei* as it relates to biofilm inhibition via the nitrate-sensing NarX-NarL two-component regulatory system. We investigated a conserved nitrate sensing system (Supplemental Fig. S1) that is important in environmental and host-associated contexts and aims to bridge a gap between these two important *B. pseudomallei* lifestyles. We characterized the *narX-narL* nitrate-sensing system in conjunction with biofilm inhibition and discovered a complex regulatory system encompassing key elements of secondary metabolism and biosynthetic gene cluster regulation, virulence, and antibiotic tolerance (Fig. 6).

Our initial observation of a biofilm inhibition phenotype that is comparable in both nitrate and nitrite treatment in oxic conditions is complicated by the fact that nitrite-dependent biofilm inhibition does not require the NarX-NarL system (Fig. 1A). This disparity lead us to hypothesize that the NarX-NarL system in *B. pseudomallei* can discriminate between nitrate and nitrite ligands, with clear preference towards nitrate, as corroborated by NarX protein characterization studies in *E. coli*^9^. A recent Tn-seq study identified the NarX-NarL TCS as a regulator of NO_3_^−^ reduction via the primary nitrate reductase NarGHJI, with the RoxS-RoxR TCS serving as a regulator of redox status and potentially NO_2_^−^ reduction in *Burkholderia thailandensis*^55^. This apparent ligand preference is further demonstrated in our transcriptomic analyses including Δ*narX* and Δ*narL* mutants in the presence of either nitrate (Figs. S3B,E) or nitrite (Figs. S3C,F). In response to nitrate, both mutants activate and upregulate 449 transcripts (7% of *B. pseudomallei* coding sequences), with 234 of those shared among them (Fig. S3B), and downregulate 353 (6% of CDS), with 184 overlapping in regulation, using stringent significance thresholds. Conversely, Δ*narX* and Δ*narL* mutants compared to the wild type in the presence of nitrite only differentially regulated 10 transcripts in either a positive or a negative manner, further suggesting that exogenous nitrite is not regulated by NarX-NarL (Fig. S3C,F). Sensing of similar N-oxides, nitrate and nitrite, are facilitated by twin two-component systems NarX-NarL and NarQ-NarP in enteric bacteria^56^, of which *B. pseudomallei* only encodes NarX-NarL^9^. Given that our previous in silico analyses did not discover a duplication of the NarX-NarL system on chromosome II in *B. pseudomallei*^5^, and given the data presented here, we propose that the NarX-NarL system preferentially binds nitrate as a ligand.

The in vitro transcriptional response to nitrosative stress provides the foundation to understand physiological responses in the context of both environmental and in vivo infection scenarios. Facultative intracellular pathogens must withstand several innate immune response molecules, such as reactive oxygen and nitrogen species, sparking an interest in potential intrinsic genetic and enzymatic resistance mechanisms in bacteria. Resistance to RNI has been shown to involve methionine and homocysteine metabolism in *M. tuberculosis* and *Salmonella typhimurium*^25,40,57^, which couples with our observation that *metE* is the most significantly upregulated locus in both nitrate-supplemented and nitrite-supplemented conditions. Further mutational analyses will be necessary to determine if transcripts identified in this study are implicated in response to RNI. To bridge this gap, we analyzed the fitness of Δ*narX* and Δ*narL B. pseudomallei* 1026b mutants in eukaryotic cell infection, and observed a significant defect in intracellular survival efficiency in both mutants (Fig. 5). *B. pseudomallei* can survive intracellularly in phagocytic cells^58^; although it is susceptible to the bactericidal activity of IFN-γ-stimulated macrophages, where RNI have a stronger effect than ROI^24^. Our observation of replication-deficient *B. pseudomallei* 1026b lacking either the NarX histidine kinase or the NarL DNA-binding regulator suggests that nitrate metabolism is important for intracellular survival. This observation parallels a similar study showing decreased, but not significant reductions in intracellular replication of a Δ*narL* mutant strain of an environmental *B. pseudomallei* strain E8, which was isolated from soil^35^. The intracellular growth deficiencies of Δ*narX* and Δ*narL B. pseudomallei* 1026b observed in our study can be explained by lack of active NarG and subsequent nitrate reductase activity, which is important for *Mycobacterium tuberculosis* intracellular growth^59^, although this definitive connection remains to be characterized in *B. pseudomallei.*

Our data also point towards a genome-wide coordination of secondary metabolite gene expression, for both characterized and cryptic biosynthetic gene clusters (BGCs) in response to nitrate and nitrite. Transcriptional activation of the complete bactobolin and malleilactone clusters as well as the cryptic cluster 16, coupled with the downregulation of the complete syrbactin biosynthesis cluster follow the same trends for either nitrate and nitrite, yet are dependent on a functioning NarX-NarL system (Fig. 4B). The expression of the cryptic cluster in nitrate-supplemented conditions will allow for characterization of the molecule that is expressed to determine its role in pathogenesis. In *B. thailandensis*, a global repressor of BGCs, MftR regulates both bactobolin and malleilactone production as well as *narX* and *narL*, and the T3SS regulator *bsaN*, suggesting that BGC regulation is inherently linked to host environment adaptation and ultimately virulence^60^. In *B. pseudomallei* bactobolin and malleilactone are quorum sensing (QS)-controlled secondary metabolites, which may have implications for the intricate QS regulation of virulence in this organism^61^. In this context, we hypothesize that nitrosative stress activates QS-regulated metabolites that can aid *B. pseudomallei* in adapting to host environments. Considering the model of BGC regulation in *B. thailandensis* where the *narX-narL* system is a key player in anerobic metabolism linked to MftR-regulated metabolite production^60^, our data reveal conditional expression of cryptic metabolite clusters under nitrate-dependent respiration.

*B. pseudomallei* can maintain infection during anaerobic conditions through nitrate sensing and denitrification^62^. In a recent genome-wide analysis of environmental and clinical strains of *B. cenocepacia*, the *narX-narL* nitrate sensing system was present in all clinically-derived strains and the *narGHJI* operon was variably present among clinical and environmental strains^63^. In *B. thailandensis*, complete denitrification is not required for anaerobic growth, yet nitrous oxide accumulation is suspected to lead to biofilm dispersal^55^. It is important to evaluate denitrification in the context of tissue-associated biofilms and intracellular infections given the relevance of NarX-NarL to environmental adaptation and regulation of virulence^63,64^. Two-component systems have been recognized as promising targets for antimicrobial drug development, given their importance for bacterial survival and pathogenicity in the host^65^. To this end, we describe the transcriptome of a clinical isolate, *B. pseudomallei* 1026b, during in vitro nitrate- and nitrite-supplemented growth conditions and characterize a signal transduction network reliant on a functional *narX-narL* two-component system. Furthermore, our investigation of this system in a cellular infection model highlights its importance as a virulence-facilitating factor in *B. pseudomallei*. Future work will continue investigations into the *narX-narL* system during infection of more complex model systems, as well as the regulation of secondary metabolite biosynthetic clusters in nitrate-rich environments.

## Methods

### Bacterial strains and growth conditions

*B. pseudomallei* 1026b, which is a model laboratory strain for research on the genetic factors that contribute to melioidosis^66^ was originally isolated from a human septicemic infection^67^. *B. pseudomallei* 1026b was grown in Lysogeny Broth (LB) media containing 10 g/L tryptone (Fisher Scientific), 5 g/L yeast extract (Becton, Dickinson and Company), and 5 g/L NaCl (Fisher Scientific) at 37 °C with aeration as described previously^5^, unless otherwise indicated. *E*. *coli* DH5α and RHO3 strains were grown in LB at 37 °C with aeration. *B. pseudomallei* experiments were carried out in biosafety level 3 (BSL-3) in the Regional Biocontainment Laboratory at Colorado State University^5^. For anaerobic growth, LB was supplemented with 0.75% glucose (LBG) and experiments were performed inside a 2.5 L anaerobic jar (AnaeroPack) using one sachet of Anaerobic Gas Generator (AnaeroPack). For experiments involving media with nitrate or nitrite, LB was supplemented with a final concentration of 10 mM sodium nitrate (Sigma) or 10 mM sodium nitrite (Matheson Coleman and Bell) as described previously^5^, unless otherwise indicated.

### Mutant strain construction and complementation

In-frame deletion mutations were constructed as previously described^43^. Briefly, *B. pseudomallei* Bp82^68^ (strain excluded from Select Agent regulations) genomic DNA was used as a template for PCR fragment generation in all experiments. DNA restriction enzymes were purchased from New England Biolabs. Primers and plasmids used for mutant strain construction and complementation are listed in Supplementary Table S4. In-frame deletion constructs were generated using Splicing Overlap Extension (SOE) PCR^69^. Flanking sequences on both sides (~ 1 Kb each) of Bp1026b_I1014 (*narX*) and Bp1026b_I1013 (*narL*) were amplified from Bp82 template DNA, and spliced via amplification of a fragment excluding the gene coding regions. Spliced overlap fragments were cloned into pEXKm5^70^ and electroporated into *E. coli* RHO3^70^. *E. coli* RHO3 pEXKm5::Δ*narX* and pEXKm5::Δ*narL* were grown in LB supplemented with diaminopimelic acid (400 µg/mL) and kanamycin (35 µg/mL) at 37 °C. Tri-parental mating with *B. pseudomallei* 1026b was facilitated with *E. coli* RHO3 pTNS3 grown in LB supplemented with ampicillin (100 µg/mL) and diaminopimelic acid (400 µg/mL). Transconjugants were selected for using kanamycin (1000 µg/mL) and X-Gluc (100 µg/mL) during screening. Merodiploids were resolved using yeast-tryptone (YT) media supplemented with 15% sucrose. In-frame deletion constructs were verified via internal and flanking primers (Supplementary Table S4 and Supplemental Fig. S5). For complementation, full-length copies of Bp1026b_I1014 and Bp1026b_I1013 were cloned into pUC18T-mini-Tn*7*T-km-LAC for IPTG-inducible expression^71^.

### Static biofilm and motility assays

Static biofilm and motility assays were performed as described previously^5,72^. All experiments were performed in LB supplemented with 10 mM sodium nitrate or 10 mM sodium nitrite unless otherwise specified, grown at 37 °C, in either aerobic or anaerobic conditions. Bacteria were cultured for 24 h for all experiments, including static biofilm and motility assays, unless otherwise specified. Biofilms were assayed in replicates of six individual wells of 96-well polystyrene plates (Nunc™ Microwell™ 96-well microplates #243656, Thermo Scientific) and processed as previously described^5^. Motility was measured by inoculating strains of interest into 0.3% semisolid LB agar, supplemented with sodium nitrate as indicated, and measuring visible diameter of bacterial spread over the specified time.

### Nitrite ion measurement

Nitrite ion (NO_2_^−^) from bacterial cultures was measured using the Griess Reagent system (Promega) following the protocol recommended by the manufacturer. Briefly, a nitrite standard reference curve was generated and included on each 96-well plate (Nunc) used for experimental samples. The Griess reaction was performed using room-temperature sulfanilamide solution and NED solution and the resulting azo compound density was measured at OD_550_. These experiments were performed in aerobic as well as anaerobic conditions with wild type, ∆*narX*, and ∆*narL* mutant strains grown statically for 24 h at 37 °C in biological triplicates and technical triplicates. Aerobic cultures were cultivated in LB media supplemented with 10 mM NaNO_3_ and anaerobic cultures were cultivated in LBG media (LB 0.75% glucose) and supplemented with 25 mM NaNO_3_.

### RNA isolation and RNA-seq library preparation

Total RNA was isolated from static-growth cultures as described previously^5^, with a few modifications. LB media was used due to previous observations of pellicle biofilm formation, in either aerobic or anaerobic conditions as indicated. Total RNA was collected form pellicle biofilms formed at the air–liquid interface from three technical replicates per biological sample grown in six-well Costar polysterene plates (Corning) for 24 h at 37 °C, at which point 1.5 mL of culture samples were collected and resuspended in RNAprotect Bacteria Reagent (Qiagen) and then QIAzol Lysis Reagent (Qiagen) before storage at − 80 °C. RNA samples were purified and depleted of genomic DNA as described previously^5^, before depletion of ribosomal RNA with Ribo-Zero rRNA Removal Kit for bacteria (Illumina) and purification using magnetic beads (AMPure). RNA-seq libraries of cDNA were generated using ScriptSeq™ Complete v2 RNA-seq Library Preparation Kit (Illumina) and purified using Monarch DNA cleanup kit (New England Biolabs). Unique barcodes were added to each sample library using ScriptSeq™ Index PCR Primers (Illumina). Libraries were analyzed on a Tapestation using HS D1000 tapes and reagents (Agilent) to determine average sizes and concentrations of the libraries. Size and molarity estimates were used to pool all libraries in equimolar concentrations. Final quality control and library quantification analyses were completed at the Microbiology, Immunology, and Pathology Next Generation Sequencing Core Facility (MIP NGS Illumina Core) at Colorado State University.

### Illumina sequencing and differential expression quantification

A NextSeq run was completed on the pooled libraries using the NextSeq 500 hi-output v2 75-cycle kit and Buffer Cartridge (Illumina). Sequence files were downloaded from the MIP NGS server, de-multiplexed according to index primers, and converted to fastq files before initial quality control using FastQC^73^. Adapter sequences were trimmed using Trimmomatic (ILLUMINACLIP:TruSeq3-SE:2:30:10 LEADING:3 TRAILING:3 SLIDINGWINDOW:4:15.

MINLEN:72) before another quality control round using FastQC. Bowtie2 was used to align sequencing reads to the reference genome GCF_000260515.1_ASM26051v1 (NCBI) and TopHat was used for transcriptome assembly. The averages for input reads and mapping efficiencies for all samples are as follows: 8,442,561.8 ± 2,610,246.1 total reads input, 7,030,147.1 ± 2,203,113.0 reads mapped or 83.2% ± 8.1% mapping efficiency overall. (Supplementary Table S5). HTseq-count (version 0.11.0) was used to count accepted hits before the DEseq2 (version 1.20.0)^36^ package was employed in R (version 3.6.1) for comprehensive differential expression analysis. Raw read count coverage values were used to compare the differential gene expression between temperature treatments, mutants, and untreated controls. Using a negative binomial distribution to estimate variance and a Bayesian approach for variance shrinkage, the DEseq2 package produced logarithmic fold-change values between the conditions tested. Wald tests were used to calculate p-value and the Benjamini–Hochberg multiple testing correction was used to correct for the false discovery rate.

### Gene expression and quantitative real-time PCR

Genomic DNA-depleted RNA samples, isolated in quadruplicate, were pooled and cDNA was synthesized using 1 µg total RNA, using the Transcriptor First Strand cDNA Synthesis kit (Roche). Primers (Supplementary Table S4) for Bp1026b_I1018 (*narG-1*), Bp1026b_II1965 (CPS III) Bp1026b_I1913 (*relA*), Bp1026b_II1245 (bactobolin), Bp1026b_II1353 (syrbactin), and Bp1026b_I0189 (*hcp-1*), were designed using the PrimerQuest tool (IDT DNA Technologies), and primer efficiencies were calculated using dilutions of cDNA samples. The 23S rRNA reference gene^74^ was used as a housekeeping control for normalization^75^, due to its consistent expression profile for all cDNA samples even after attempted ribodepletion. qRT-PCR was performed using 10 ng total cDNA and the same cycling conditions described previously^5^. Relative transcript abundance was measured using the Pfaffl method^76^.

### Infection of murine macrophage cells

Murine macrophage cells (RAW 264.7 cell line, ATCC) were propagated in Dulbecco’s Modified Eagle Medium (DMEM, Gibco) supplemented with 10% fetal bovine serum and growth at 37 °C with 5% CO_2_ and 80–90% relative humidity in T-75 flasks (Corning). *B. pseudomallei* 1026b strains were grown overnight at 37 °C with aeration to stationary phase and diluted to an appropriate OD_600_ for an infection inoculum of ~ 1 × 10^6^ CFU/mL. RAW cells were seeded in 12-well cell culture dishes (Corning) at a density of ~ 5 × 10^5^ cells. Bacteria were resuspended in DMEM and added to RAW cells at a MOI of ~ 2 and incubated at 37 °C for 1 h. RAW cells were washed with 1X PBS and culture media was replaced with fresh media supplemented with kanamycin (750 µg/mL) before a 2-h incubation at 37 °C to kill extracellular bacteria. RAW cells were washed and lysed in 1 × PBS containing 0.2% Triton X-100 for 2.5 min and serially diluted and plated on LB agar to quantify intracellular bacteria. Survival of the wild type, Δ*narX*, and Δ*narL* strains were analyzed in macrophage using the same infection procedure above. RAW 264.7 cells were incubated with bacteria at MOI of ~ 2 for 20 h at 37 °C and sampled at 6 h and 20 h. Cells were washed, lysed and serially diluted to plate for CFU/mL as described above.

## Supplementary Information

Supplementary Figure 1.

Supplementary Figure 2.

Supplementary Figure 3.

Supplementary Figure 4.

Supplementary Figure 5.

Supplementary Table 1.

Supplementary Table 2.

Supplementary Table 3.

Supplementary Table 4.

Supplementary Table 5.

Supplementary Legends.

## Data Availability

Raw sequence files associated with this study are deposited at the European Nucleotide Archive (http://www.ebi.ac.uk/ena) under the primary accession number PRJEB38907.
