# Supplementary figures and images for: The NarX-NarL two-component system regulates biofilm formation, natural product biosynthesis, and host-associated survival in Burkholderia pseudomallei
Source: Sci Rep. 2022 Jan 7;12:203. doi: 10.1038/s41598-021-04053-6 (PMC8742066; doi:10.1038/s41598-021-04053-6)

# Supplemental Figure 1

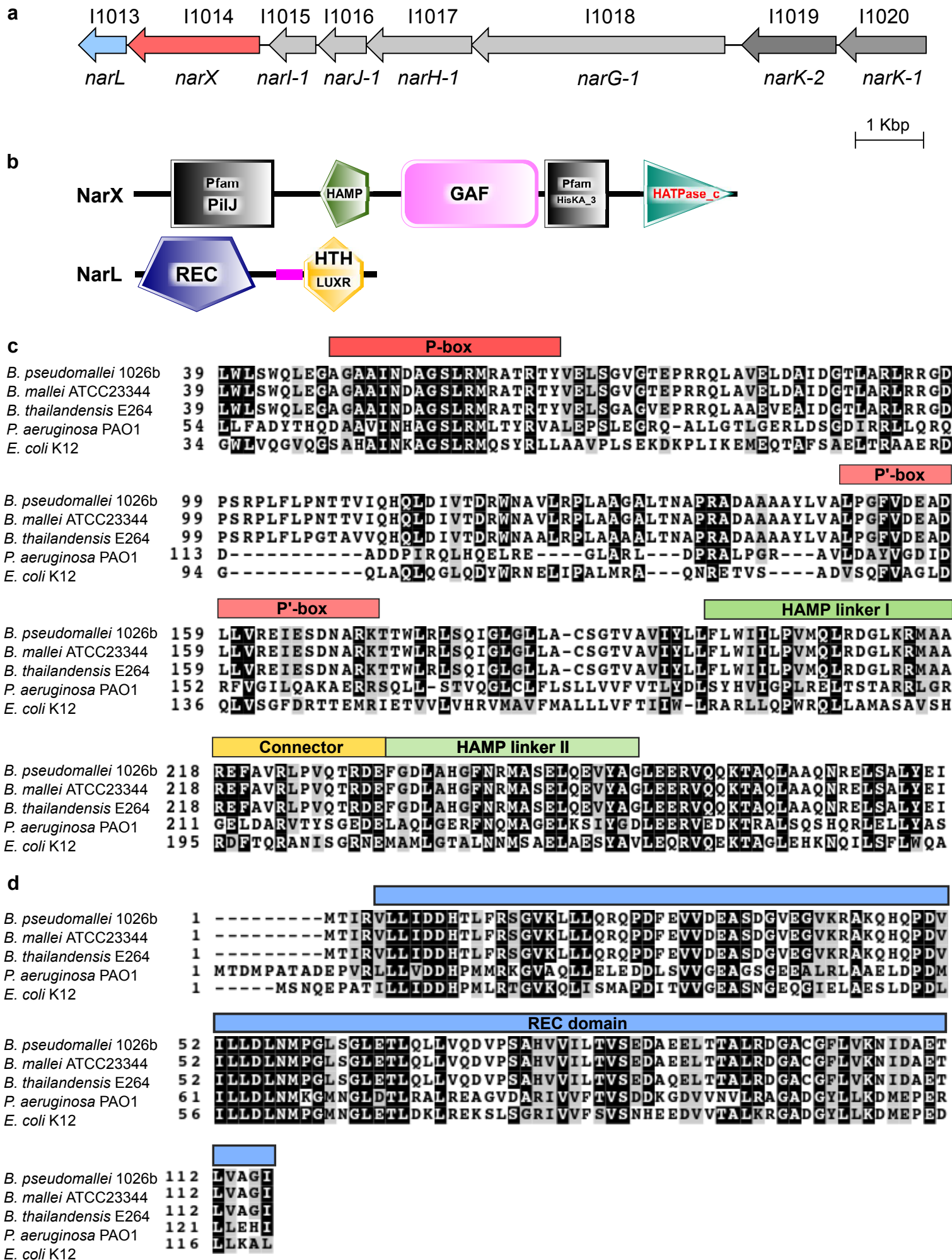

Supplement: Supplementary file 1 — Supplementary Figure 1. [file 41598_2021_4053_MOESM1_ESM.pdf]

Supplemental Figure 2

*B. pseudomallei* 1026b

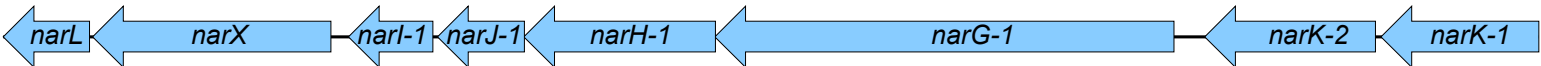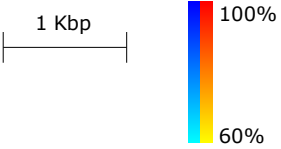

*B. mallei* ATCC 24433

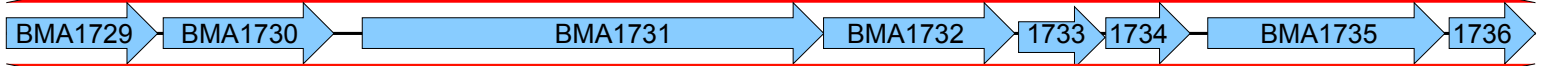

*B. thailandensis* E264

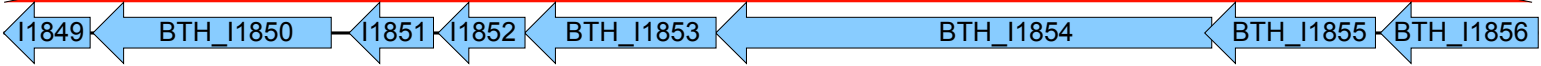

Supplement: Supplementary file 2 — Supplementary Figure 2. [file 41598_2021_4053_MOESM2_ESM.pdf]

Supplemental Figure 3

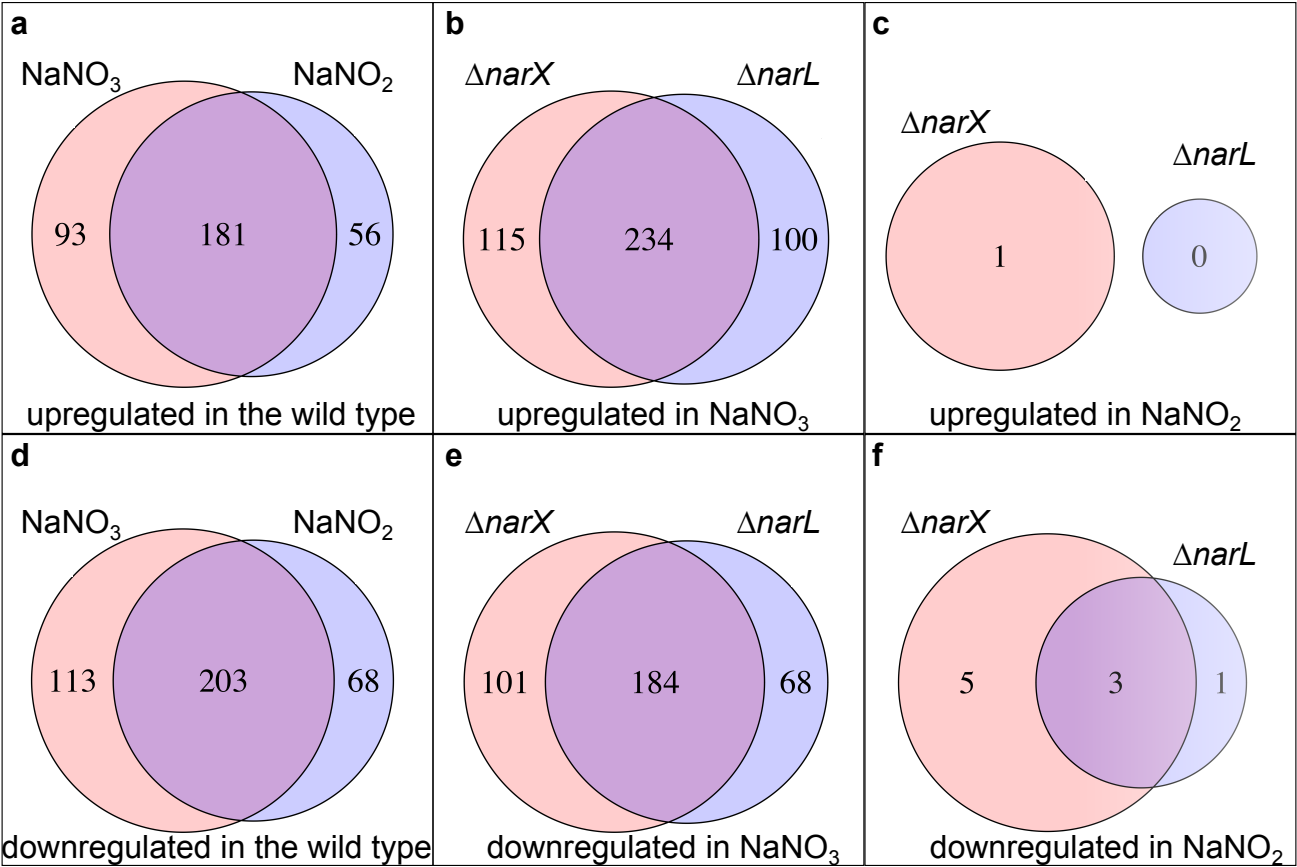

Supplement: Supplementary file 3 — Supplementary Figure 3. [file 41598_2021_4053_MOESM3_ESM.pdf]

Supplemental Figure 4

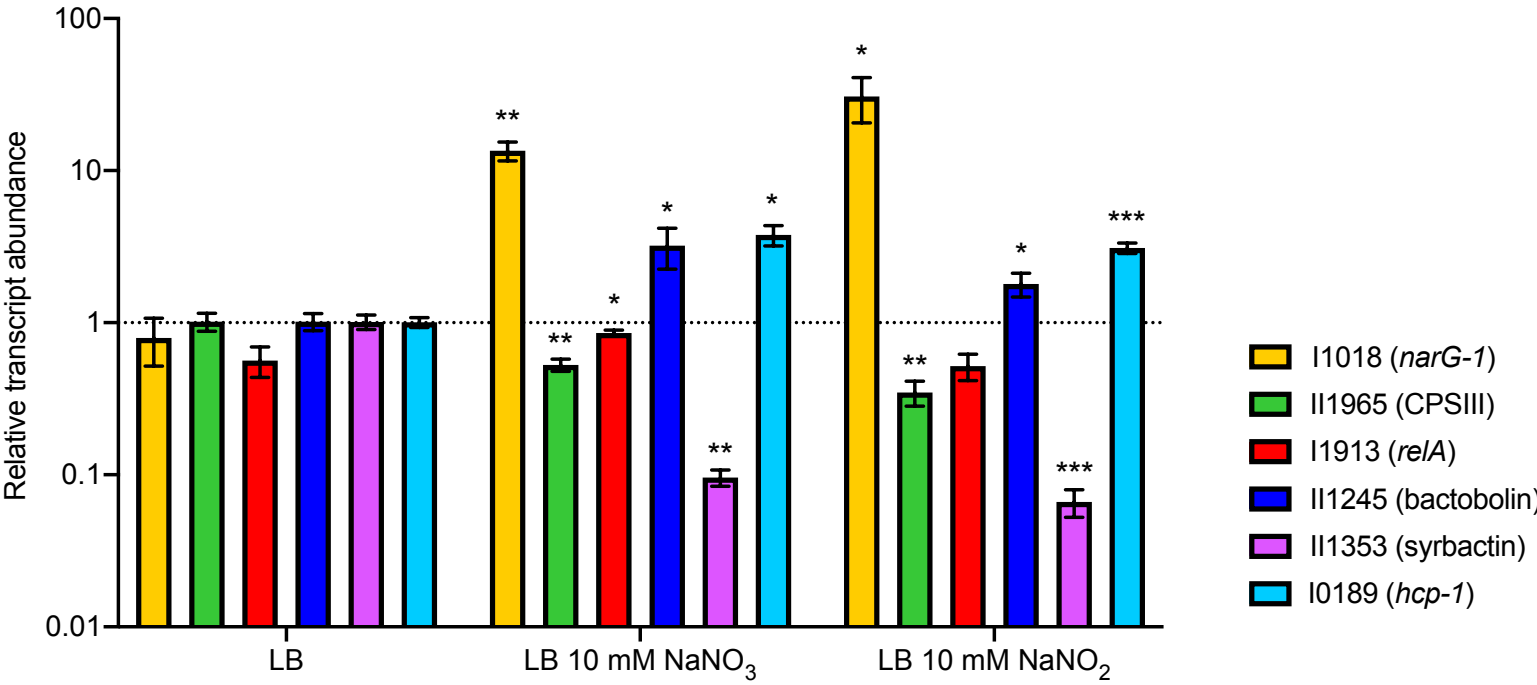

Supplement: Supplementary file 4 — Supplementary Figure 4. [file 41598_2021_4053_MOESM4_ESM.pdf]

Supplemental Figure 5

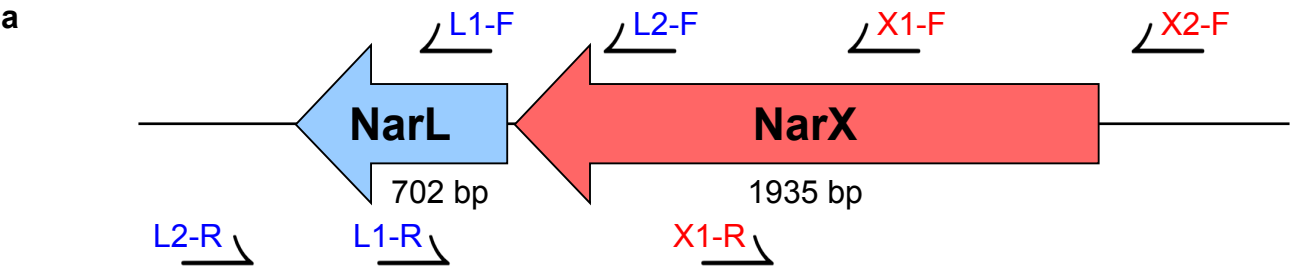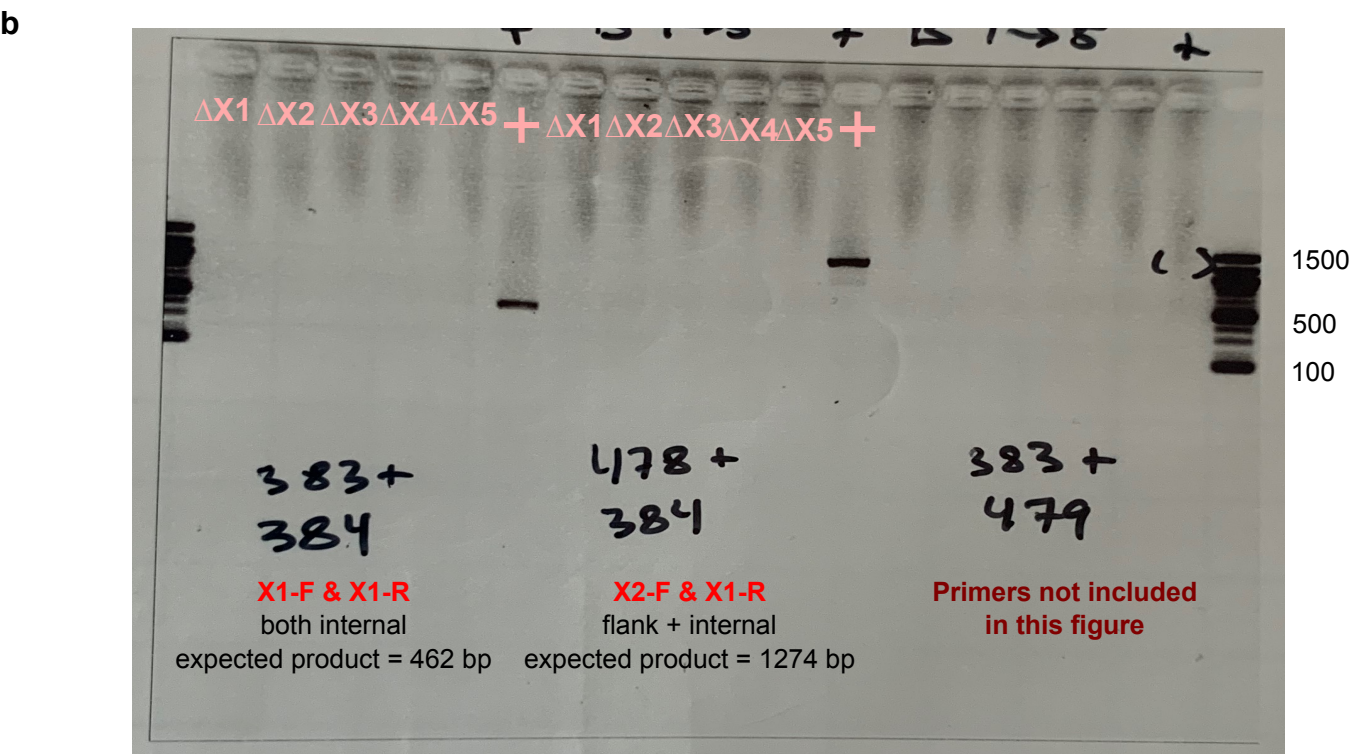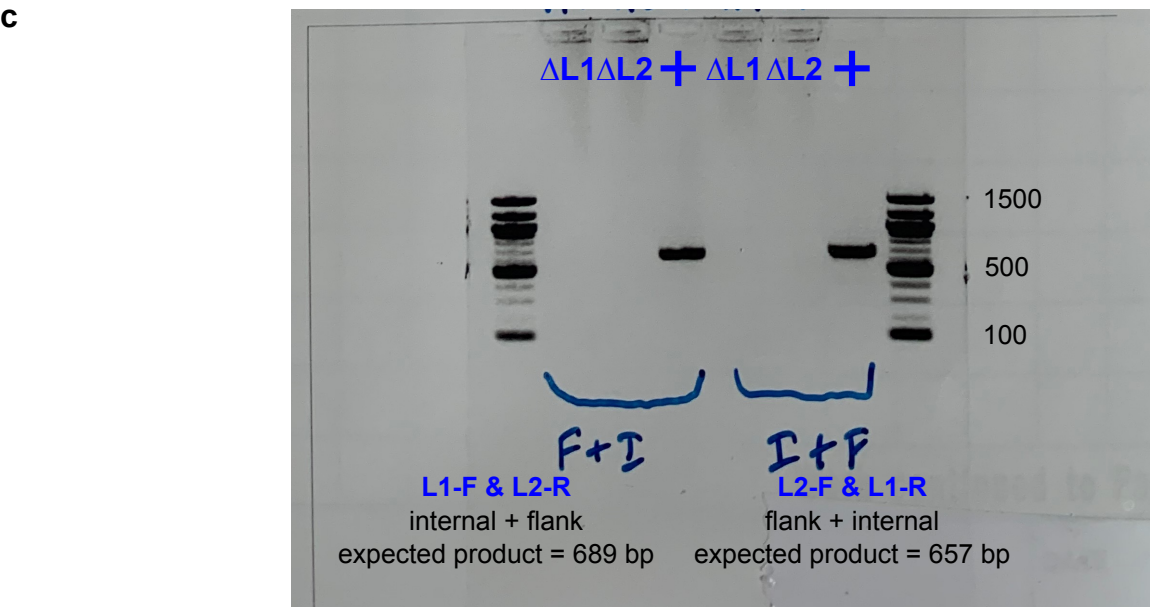

Supplement: Supplementary file 5 — Supplementary Figure 5. [file 41598_2021_4053_MOESM5_ESM.pdf]
