# Supplementary material for: The NarX-NarL two-component system regulates biofilm formation, natural product biosynthesis, and host-associated survival in Burkholderia pseudomallei
Source: Sci Rep. 2022 Jan 7;12:203. doi: 10.1038/s41598-021-04053-6 (PMC8742066; doi:10.1038/s41598-021-04053-6)
Supplement: Supplementary file 6 — Supplementary Table 1. [file 41598_2021_4053_MOESM6_ESM.docx]

| ID | No. Genes | Genes in Cluster | Log2 FC Mean | Log2 FC SD | Expression Trend |
| --- | --- | --- | --- | --- | --- |
| 1 | 4 | I0001, I0002, I0003, I0004 | -0.9207 | 0.2024 | Down |
| 2 | 11 | I0180, I0181, I0182, I0183, I0184, I0185, I0186, I0187, I0188, I0189, I0190 | 0.5888 | 0.0309 | Up |
| 3 | 8 | I0202, I0203, I0204, I0205, I0206, I0207, I0208, I0209 | -0.8623 | 0.0823 | Down |
| 4 | 7 | I0449, I0450, I0451, I0452, I0453, I0454, I0455 | -0.7196 | 0.0333 | Down |
| 5 | 5 | I0506, I0507, I0509, I0508, I0510 | 0.5304 | 0.0081 | Up |
| 6 | 10 | I0597, I0598, I0599, I0600, I0601, I0602, I0603, I0604, I0605, I0606 | -0.9851 | 0.1576 | Down |
| 7 | 12 | I0703, I0704, I0705, I0706, I0707, I0708, I0709, I0710, I0711, I0712, I0713, I0714 | 0.7376 | 0.1338 | Up |
| 8 | 11 | I1012, I1013, I1014, I1015, I1016, I1017, I1018, I1019, I1020, I1021, I1023 | 1.475 | 0.6393 | Up |
| 9 | 7 | I1162, I1163, I1164, I1165, I1166, I1167, I1168 | 0.5481 | 0.0204 | Up |
| 10 | 11 | I1173, I1172, I1174, I1175, I1176, I1177, I1178, I1179, I1180, I1182, I1183 | 0.6515 | 0.0872 | Up |
| 11 | 10 | I1202, I1203, I1204, I1205, I1206, I1207, I1208, I1209, I1211, I1212 | -0.9315 | 0.1395 | Down |
| 12 | 11 | I1725, I1726, I1727, I1728, I1729, I1730, I1731, I1732, I1733, I1734, I1735 | 0.6804 | 0.0494 | Up |
| 13 | 15 | I2017, I2018, I2019, I2020, I2021, I2022, I2023, I2024, I2025, I2026, I2027, I2028, I2029, I2030, I2031 | 0.6847 | 0.0724 | Up |
| 14 | 15 | I2133, I2134, I2135, I2136, I2137, I2138, I2139, I2140, I2141, I2142, I2145, I2146, I2148, I2149, I2150 | 0.6509 | 0.0806 | Up |
| 15 | 8 | I2159, I2160, I2161, I2163, I2165, I2167, I2168, I2169 | 0.5869 | 0.0531 | Up |
| 16 | 13 | I2282, I2283, I2284, I2285, I2286, I2287, I2288, I2289, I2290, I2291, I2292, I2293, I2294 | 1.3565 | 0.4006 | Up |
| 17 | 3 | I2330, I2331, I2332 | 0.5236 | 0.0017 | Up |
| 18 | 11 | I2467, I2468, I2469, I2470, I2471, I2473, I2474, I2475, I2476, I2479, I2480 | -1.2768 | 0.2544 | Down |
| 19 | 3 | I2626, I2627, I2628 | -0.6907 | 0.0096 | Down |
| 20 | 2 | I2695, I2696 | 0.523 | 0.0042 | Up |
| 21 | 13 | I2716, I2718, I2719, I2720, I2721, I2722, I2723, I2724, I2725, I2726, I2727, I2728, I2729 | 0.7016 | 0.0889 | Up |
| 22 | 13 | I2914, I2915, I2916, I2918, I2917, I2919, I2920, I2921, I2922, I2923, I2924, I2925, I2926 | -1.274 | 0.3835 | Down |
| 23 | 3 | I2930, I2931, I2932 | 0.5643 | 0.003 | Up |
| 24 | 4 | I3001, I3002, I3003, I3004 | -0.7224 | 0.0377 | Down |
| 25 | 27 | I3006, I3007, I3008, I3009, I3010, I3011, I3012, I3013, I3014, I3015, I3016, I3017, I3018, I3019, I3020, I3021, I3022, I3024, I3023, I3025, I3026, I3027, I3028, I3029, I3030, I3031, I3032 | -1.2112 | 0.2934 | Down |
| 26 | 5 | I3183, I3184, I3185, I3186, I3187 | -0.6895 | 0.0187 | Down |
| 27 | 40 | I3419, I3420, I3421, I3422, I3423, I3424, I3425, I3426, I3427, I3428, I3429, I3430, I3431, I3432, I3433, I3434, I3435, I3436, I3437, I3438, I3439, I3440, I3441, I3442, I3443, I3444, I3445, I3446, I3447, I3448, I3449, I3450, I3451, I3453, I3454, I3455, I3456, I3457, I3458, I3460 | -0.927 | 0.102 | Down |

**S1 Table. Expression trends for differentially regulated transcripts on Chromosome I in response to 10 mM NaNO_3_**
