# Supplementary material for: The NarX-NarL two-component system regulates biofilm formation, natural product biosynthesis, and host-associated survival in Burkholderia pseudomallei
Source: Sci Rep. 2022 Jan 7;12:203. doi: 10.1038/s41598-021-04053-6 (PMC8742066; doi:10.1038/s41598-021-04053-6)
Supplement: Supplementary file 7 — Supplementary Table 2. [file 41598_2021_4053_MOESM7_ESM.docx]

| ID | No. Genes | Genes in Cluster | Log2 FC Mean | Log2 FC SD | Expression Trend |
| --- | --- | --- | --- | --- | --- |
| 1 | 3 | II0199, II0201, II0200 | -0.9365 | 0.011 | Down |
| 2 | 4 | II0232, II0233, II0234, II0235 | -0.9472 | 0.0139 | Down |
| 3 | 5 | II0336, II0337, II0338, II0339, II0340 | 1.1898 | 0.0388 | Up |
| 4 | 4 | II0639, II0640, II0641, II0642 | 1.1602 | 0.0365 | Up |
| 5 | 4 | II1137, II1138, II1139, II1140 | 1.3533 | 0.1301 | Up |
| 6 | 7 | II1231, II1232, II1233, II1234, II1235, II1236, II1237 | 1.3228 | 0.0763 | Up |
| 7 | 14 | II1241, II1242, II1243, II1244, II1245, II1246, II1247, II1248, II1249, II1251, II1250, II1252, II1253, II1254 | 1.6048 | 0.1707 | Up |
| 8 | 3 | II1261, II1262, II1263 | -1.0715 | 0.0498 | Down |
| 9 | 14 | II1342, II1343, II1344, II1345, II1346, II1347, II1348, II1349, II1350, II1351, II1352, II1353, II1354, II1355 | -2.0991 | 0.6582 | Down |
| 10 | 6 | II1414, II1415, II1416, II1417, II1418, II1419 | -1.0476 | 0.0779 | Down |
| 11 | 9 | II1447, II1448, II1449, II1450, II1451, II1452, II1454, II1455, II1456 | -1.1587 | 0.0947 | Down |
| 12 | 29 | II1612, II1615, II1616, II1617, II1618, II1619, II1620, II1621, II1622, II1623, II1624, II1625, II1626, II1627, II1628, II1629, II1630, II1631, II1632, II1633, II1634, II1635, II1636, II1637, II1638, II1639, II1640, II1641, II1642 | -1.1798 | 0.1661 | Down |
| 13 | 6 | II1799, II1800, II1801, II1802, II1803, II1804 | -1.0501 | 0.06 | Down |
| 14 | 13 | II1931, II1932, II1933, II1934, II1935, II1936, II1937, II1938, II1939, II1940, II1941, II1942, II1943 | 1.8051 | 0.3416 | Up |
| 15 | 6 | II1959, II1960, II1961, II1962, II1963, II1964 | -0.9653 | 0.0242 | Down |
| 16 | 5 | II2030, II2031, II2032, II2033, II2034 | -1.0977 | 0.0339 | Down |
| 17 | 8 | II2043, II2042, II2044, II2045, II2046, II2047, II2048, II2049 | -1.1748 | 0.0985 | Down |
| 18 | 30 | II2068, II2069, II2070, II2071, II2072, II2073, II2074, II2075, II2076, II2077, II2078, II2079, II2080, II2082, II2081, II2083, II2084, II2085, II2086, II2087, II2088, II2089, II2090, II2091, II2092, II2093, II2094, II2095, II2096, II2097 | 1.3065 | 0.1232 | Up |
| 19 | 8 | II2141, II2142, II2144 II2145, II2146, II2147, II2148, II2149 | 1.6808 | 0.3017 | Up |
| 20 | 5 | II2418, II2419, II2420, II2421, II2422 | -1.0116 | 0.0618 | Down |
| 21 | 13 | II2437, II2438, II2439, II2440, II2441, II2442, II2443, II2444, II2445, II2446, II2447, II2449, II2448 | 1.3985 | 0.1535 | Up |

**S2 Table. Differentially regulated gene clusters on Chromosome II in response to 10 mM NaNO_3_**
