# Supplementary material for: The NarX-NarL two-component system regulates biofilm formation, natural product biosynthesis, and host-associated survival in Burkholderia pseudomallei
Source: Sci Rep. 2022 Jan 7;12:203. doi: 10.1038/s41598-021-04053-6 (PMC8742066; doi:10.1038/s41598-021-04053-6)
Supplement: Supplementary file 9 — Supplementary Table 4. [file 41598_2021_4053_MOESM9_ESM.docx]

| Primers | Sequence (5’ – 3’) or Notes | Use | Ref |
| --- | --- | --- | --- |
| Δ*narX* A | NNNCCCGGGTCATGGATTATCTGAATACG | SOE Δ*narX* fragment 1 | This study |
| Δ*narX* B | CTATGCCGCCTGTCGCGCGTCGTCGGGGGAGGGCGGGAGCCAT | SOE Δ*narX* fragment 1 | This study |
| Δ*narX* C | ATGGCTCCCGCCCTCCCCGACGACGCGCGACAGGCGGCATAG | SOE Δ*narX* fragment 2 | This study |
| Δ*narX* D | NNNGAATTCGACGCCGTTGTAGGTTTTCT | SOE Δ*narX* fragment 2 | This study |
| Δ*narL* A | NNNCCCGGGAGGACAATTGTCATGT | SOE Δ*narX* fragment 1 | This study |
| Δ*narL* B | TTATGCCTCGGCCGGATGCGAACAGTACCCGTATGGTCAT | SOE Δ*narX* fragment 1 | This study |
| Δ*narL* C | ATGACCATACGGGTACTGTTCGCATCCGGCCGAGGCATAA | SOE Δ*narL* fragment 2 | This study |
| Δ*narL* D | NNNGAATTCCAGTTCTATCGCGTG | SOE Δ*narL* fragment 2 | This study |
| *narX* FWD | NNNCCCGGGATGGCTCCCGCCCTCCCCGACT | Complementing Δ*narX* | [5] |
| *narX* REV | NNNAAGCTTCTATGCCGCCTGTCGCGCGT | Complementing Δ*narX* | [5] |
| *narL* FWD | NNNCCCGGGATGACCATACGGGTACTGTT | Complementing Δ*narL* | [5] |
| *narL* REV | NNNAAGCTTTTATGCCTCGGCCGGATGCG | Complementing Δ*narL* | [5] |
| X1-F | GCGAATTGCAGGAGGTGTA | Internal deletion check | This study |
| X1-R | GATAGTGCAGCGAGAACGAG | Internal deletion check | This study |
| X2-F | ATGCCTCACGTGCTCGAATGTC | Flank deletion check | This study |
| L1-F | GTGATCCTGCTCGATCTGAACAT | Internal deletion check | This study |
| L1-R | GTCAACTGGTCGGGATCGAC | Internal deletion check | This study |
| L2-F | TGTCGGGCTCACGATCATGC | Flank deletion check | This study |
| L2-R | ATGCGTGAGGCCGACATCTTAC | Flank deletion check | This study |
| I1018 FWD | CGAGATGGTGAAATTCG | *narG-1* qPCR | This study |
| I1018 REV | TACTGCTTCACGTAGTC | *narG-1* qPCR | This study |
| II1965 FWD | CAGATTCACCGGATCGTCAC | CPSIII qPCR | This study |
| II1965 REV | CTCGATGACCTCCTGATTGAAG | CPSIII qPCR | This study |
| I1913 FWD | CGGACATCAAGGATTGCTACAC | *relA* qPCR | This study |
| I1913 REV | GACCGTATGCAGCGATTTGT | *relA* qPCR | This study |
| II1245 FWD | GACGCTCGGCTACGAATAC | Bactobolin qPCR | This study |
| II1245 REV | GGGATAGTTGGACACCATGAG | Bactobolin qPCR | This study |
| II1353 FWD | ATTACGCGGCCACGATTAC | Syr qPCR | This study |
| II1353 REV | CGTTCAACGCGACCTTCA | Syr qPCR | This study |
| I0189 FWD | ACGAAGTGACGATCGATTTCC | *hcp-1* qPCR | This study |
| I0189 REV | GATCACGTACTTCAGCTTGATCT | *hcp-1* qPCR | This study |
| II0386 FWD | GTAGACCCGAAACCAGGTGA | 23S qPCR | [72] |
| II0386 REV | CACCCCTATCCACAGCTCAT | 23S qPCR | [72] |

**S4 Table. Primers used in this study for in-frame deletions, complementation, and quantitative real-time PCR.**
