# Supplementary material for: The NarX-NarL two-component system regulates biofilm formation, natural product biosynthesis, and host-associated survival in Burkholderia pseudomallei
Source: Sci Rep. 2022 Jan 7;12:203. doi: 10.1038/s41598-021-04053-6 (PMC8742066; doi:10.1038/s41598-021-04053-6)
Supplement: Supplementary file 11 — Supplementary Legends. [file 41598_2021_4053_MOESM11_ESM.docx]

**Supplemental Figure 1. Orientation and conservation of the *narXL-narGHJI_1_-narK_2_narK_1_* cluster.** (A) Genomic orientation of the *narX-narL* regulatory system (red and blue, respectively), *narGHJI-1* dissimilatory nitrate reductase (light grey), and *narK-2* and *narK-1* nitrate/nitrite transporters (dark grey) in *B. pseudomallei* 1026b (coding sequences to scale). (B) Simple Modular Architecture Research Tool (SMART) protein domain analysis of NarX (Bp1026b_I1014) and NarL (Bp1026b_I1013). (C) Amino acid conservation of key residues in the sensory module of NarX (Bp1026b_I1014), including periplasmic domain sequences (P and P’ boxes) and HAMP (linker I, linker II, and connector) linker elements of *B. pseudomallei* 1026b, *B. mallei* ATCC 23344, *B. thailandensis* E264, *P. aeruginosa* PAO1, and *E. coli* K12. (D) Amino acid conservation of key residues of the receiver domain of NarL (Bp1026b_I1013) including the same organisms as above. Multiple sequence alignments were generated using Clustal Omega and visualized using BoxShade v3.2 where black boxes indicate identical residues and grey boxes indicate similar sequences.

**Supplemental Figure 2. Genomic conservation of the Nar regulon in the Bpc.** *Burkholderia pseudomallei* 1026b*, B. mallei* ATCC23344*,* and *B. thailandensis* E264 nitrate reduction regulons are compared here. Orthologous sequences were extracted and aligned using EasyFig [75] in Python v2.7. Illustrations depict results of blastn annotations represented by colored bars spanning chromosomal segments, with minimum percent identity of 0.60 and a threshold E-value of 1E-3. Yellow-to-red bars depict sequence homology amid inverted sequences on a color density gradient indicating percent homology, whereby dark red indicates the most homology.

**Supplemental Figure 3. Nitrate and nitrite treatments differentially regulate similar transcripts in wild-type *B. pseudomallei,* while *narX* and *narL* mutants respond to nitrate but not nitrite treatments.** (**a**, **d**) Wild-type *B. pseudomallei* in either the nitrate (NO_3_^-^) or nitrite (NO_2_^-^) treatment condition. (**a**) Upregulated transcripts: nitrate vs. LB = 274, nitrite vs LB = 237; (**d**) downregulated transcripts: nitrate vs. LB = 316, nitrite vs LB = 239. (**b**, **e**) Δ*narX* and Δ*narL* strains in the nitrate (NO_3_^-^) treatment condition. (**b**) Upregulated transcripts: Δ*narX* vs. wild type = 349, Δ*narL* vs. wild type = 334; (**e**) downregulated transcripts: Δ*narX* vs. wild type = 285, Δ*narL* vs. wild type = 252. (**c**, **f**) Δ*narX* and Δ*narL* strains in the nitrite (NO_2_^-^) treatment condition; (**c**) upregulated transcripts: Δ*narX* vs. wild type = 1, Δ*narL* vs. wild type = 0; (**f**) downregulated transcripts: Δ*narX* vs. wild type = 8, Δ*narL* vs. wild type = 4.

**Supplemental Figure 4. Quantitative evaluation of key transcripts confirms trends in the DESeq2 data set.** Relative abundance of transcripts differentially regulated by both nitrate and nitrite treatment conditions as compared to baseline expression in LB. Fold change in transcript level was calculated using the Pfaffl method and normalized to the housekeeping transcript for 23S rRNA. Statistical significance was determined using a one-tailed heteroscedastic Student’s T-test (* = p<0.05, ** = p<0.01, *** = p<0.001).

**Supplemental Figure 5. Validation of deletion mutant strains 1026b Δ*narX* and Δ*narL*.** Mutant deletion constructs were generated using Splicing Overlap Extension (SOE) PCR as described in the “Mutant strain construction and complementation” Methods section. (**a**) Schematic diagram of genetic orientation of NarX and NarL coding sequences, their length in bp, and the 8 internal and flanking primers used to validate deletions (Supplementary Table S4). (**b**) Agarose gel after electrophoresis including 3 distinct primer pairs (X1-F & X1-R both internal, X2-F & X1-R flank and internal, X1-F & X2-R internal and flank) with their expected product sizes. 5 individual mutants were tested (ΔX1-5) as well as a positive control using *B. pseudomallei* Bp82 genomic DNA. (**c**) Two distinct primer pairs were used (L1-F & L2-R internal and flank, L2-F & L1-R flank and internal) in 2 individual mutants (ΔL1-2). A 100bp ladder was used, indicated by 1500bp, 500bp, and 100bp demarcations, as reference for positive controls and deletion mutants.

**Supplementary Table S1. Expression trends for differentially regulated transcripts on Chromosome I in response to 10 mM NaNO_3_.** Values output from the Webserver for Position Related data analysis of gene Expression in Prokaryotes (WoPPER) showing cluster IDs for similarly regulated transcripts on Chromosome I of *B. pseudomallei* 1026b, total genes associated with each cluster, the gene names in each cluster, the mean Log2 fold change, Log2 fold change standard deviation, and the general expression trend for each cluster.

**Supplementary Table S2. Differentially regulated gene clusters on Chromosome II in response to 10 mM NaNO_3_.** Values output from the Webserver for Position Related data analysis of gene Expression in Prokaryotes (WoPPER) showing cluster IDs for similarly regulated transcripts on Chromosome II of *B. pseudomallei* 1026b, total genes associated with each cluster, the gene names in each cluster, the mean Log2 fold change, Log2 fold change standard deviation, and the general expression trend for each cluster.

**Supplementary Table S3. Fold changes for all *B. pseudomallei* 1026b transcripts for all pairwise comparisons analyzed via DESeq2.** Provided here are all transcripts for which have a baseMean value higher than 0, which amount to at least 98% of all *B. pseudomallei* 1026b transcripts in all 6 conditions tested. Pairwise comparisons of differential expression (fold change) are represented on 6 individual tabs in this spreadsheet. BaseMean was determined by the DESeq2 algorithm (the average of the normalized count values, dividing by size factors, taken over all samples in the DESeqDataSet). Loci from *B. pseudomallei* 1026b and their annotations are provided to facilitate searching of transcripts. Log2FoldChange is a direct output from the DESeq2 algorithm and the Fold Change was calculated for the reader’s reference. LfcSE is the standard error of the log2FoldChange. Stat is the Wald test statistic, the log2FoldChange value divided by lfcSE, which is used to generate the pvalue in comparison to a normal distribution. Padj is the adjusted p-value using the Benjamini-Hochberg multiple testing correction was used to correct for the false discovery rate.

**Supplementary Table S4. Primers used in this study for in-frame deletions, complementation, and quantitative real-time PCR.** A list of all primers used in this study.

**Supplementary Table S5.** **TopHat mapping efficiencies for all samples used for RNAseq analyses.** All individual RNA sequencing libraries generated in this study, their sample IDs, total number of reads generated for mapping to the reference *B. pseudomallei* 1026b reference genome, the total number of reads mapped, and the mapping efficiency using the TopHat algorithm.
